# Pan‐Variant SARS‐CoV‐2 Vaccines Induce Protective Immunity by Targeting Conserved Epitopes

**DOI:** 10.1002/advs.202409919

**Published:** 2025-02-27

**Authors:** Masaud Shah, Sung Ung Moon, Ji‐Yon Shin, Ji‐Hye Choi, Doyoon Kim, Hyun Goo Woo

**Affiliations:** ^1^ Department of Physiology Ajou University School of Medicine Suwon 16499 Republic of Korea; ^2^ Ajou Translational Omics Center (ATOC) Research Institute for Innovative Medicine Ajou University Medical Center Suwon 16499 Republic of Korea; ^3^ AI‐Superconvergence KIURI Translational Research Center Ajou University School of Medicine Suwon 16499 Republic of Korea; ^4^ Department of Biomedical Science Graduate School Ajou University Suwon 16499 Republic of Korea

**Keywords:** MHC, multiepitope vaccine, neutralization, pan‐variant, peptides, SARS‐CoV‐2, vaccine

## Abstract

The development of a globally effective COVID‐19 vaccine faces significant challenges, particularly in redirecting the B‐cell response from immunodominant yet variable regions of viral proteins toward their conserved domains. To address this, an integrated strategy is implemented that combines classical B‐cell epitope prediction with protein‐antibody cluster docking and antibody titer analysis from 30 vaccinated and convalescent individuals. This approach yields stable immunodominant and immunoprevalent B‐cell epitopes capable of eliciting robust antibody responses in BALB/c mice and effectively neutralizing pseudoviruses expressing the Spike protein of SARS‐CoV‐2 variants of concern, including Alpha, Beta, Gamma, Delta, and Omicron. To achieve a broader T‐cell‐based immune response, promiscuous T‐cell epitopes are identified by integrating classical T‐cell epitope predictions, differential scanning fluorimetry, and peptide‐MHC structural analysis. Unique peptides with conserved MHC‐anchoring residues are identified, enabling binding to a spectrum of MHC‐I and MHC‐II haplotypes. These peptides elicit strong interferon gamma responses in human peripheral blood mononuclear cells and demonstrate cross‐species efficacy by activating both CD4+ and CD8+ T‐cells in BALB/c mice. Collectively, these findings highlight the significance of innovative vaccine strategies targeting immunodominant/immunoprevalent B‐cell and promiscuous T‐cell epitopes to drive broad and robust humoral and cellular immune responses against a wide range of SARS‐CoV‐2 variants.

## Graphical abstract

An integrative approach identifies conserved B‐cell and T‐cell epitopes within SARS‐CoV‐2 proteins, redirecting immune responses from variable to conserved regions. These epitopes elicit robust humoral and cellular immunity, neutralizing diverse viral variants. Promiscuous T‐cell epitopes demonstrate cross‐species efficacy, highlighting their potential in universal COVID‐19 vaccine design against evolving SARS‐CoV‐2 variants.

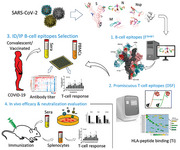

## 1 Introduction

The continuous emergence of new SARS‐CoV‐2 variants poses a significant challenge in developing globally effective vaccines and achieving sufficient immunization to contain the virus.^[^ ^1^, ^2^ ^]^ The Spike protein, which remained the primary target for COVID‐19 vaccine development, contains hotspots of mutations within its hypervariable receptor‐binding domain (RBD) that are directly involved in both vaccines‐ and infection‐induced immune escape and in host susceptibility.^[^ ^3^ ^]^ While both vaccines and natural infection, elicit neutralizing antibodies targeting multiple domains of the Spike, over 90% of these antibodies are directed against RBD, underscoring its immunodominance.^[^ ^4^ ^]^ However, this immunodominance, combined with the RBD's variability, skews the immune response toward predominantly variable epitopes, thereby limiting the breadth of protection. Antibodies targeting conserved regions of the Spike protein could provide cross‐protection against diverse variants,^[^ ^5^ ^]^ but they are often overshadowed by the RBD‐focused response. To address these limitations, strategies like immunofocusing,^[^ ^6^ ^]^ which redirect the humoral immune response from subtype‐specific variable immunodominant (ID) epitopes to conserved regions, are critical for developing broadly effective vaccines.^[^ ^7^ ^]^ This has been exemplified by the identification of broad‐spectrum neutralizing antibodies targeting the fusion peptide (FP) and stem helix (SH) of the SARS‐CoV‐2 Spike.^[^ ^8^, ^9^ ^]^ Besides, immunofocusing combined with multi‐epitope‐based vaccine strategies has facilitated the development of potent peptide vaccines, offering tailored protection against COVID‐19.^[^ ^10^, ^11^, ^12^ ^]^

In the context of B‐cell responses, ID reflects the strength of antigen recognition by B‐cell receptors (BCRs), while immunoprevalence (IP) refers to the frequency of antigen recognition by a given BCR‐antigen pair.^[^ ^13^ ^]^ Together, ID and IP shape the magnitude and breadth of the antibody response against antigens in the host. These attributes are influenced by factors such as the antigen's transcription and expression levels, structural stability, and expression patterns across various cell types or anatomical sites.^[^ ^14^ ^]^ In the context of T cells, promiscuity refers to a peptide's ability to bind multiple human leukocyte antigen (HLA) allelic variants (HLA supertypes), particularly when these variants are structurally or genetically related.^[^ ^15^ ^]^ Consequently, a peptide‐major histocompatibility complex (MHC) complex (pMHC) can be recognized by several T‐cell receptors (TCRs), usually sharing a sequence similarity pattern.^[^ ^16^ ^]^ Promiscuous T‐cell epitopes also display strong ID and IP characteristics;^[^ ^17^ ^]^ nonetheless, this study explicitly used the ID and IP notions for B‐cell epitopes and promiscuous notions for T‐cell epitopes.

To steer the B‐cell response toward conserved yet ID epitopes, we performed clustered docking of SARS‐CoV‐2 neutralizing antibodies (nAbs) onto its structural proteins, isolating both linear and conformational epitopes. Variations in B‐cell responses to these epitopes were validated by analyzing antibody titers in sera from COVID‐19 vaccinated and convalescent individuals. The identified ID and IP B‐cell epitopes were further characterized based on their antibody titers and evaluated for their ability to elicit robust antibody responses in vivo. In the context of T‐cells, although several studies have identified HLA‐bound peptides, they often fall short of confirming whether these epitopes can effectively recognize and activate TCRs—a crucial determinant of the immunogenicity of potential antigenic peptides.^[^ ^18^ ^]^ While HLA binding is necessary for T‐cell recognition, it is not sufficient, as it neither guarantees peptide generation through antigen processing nor ensures the presence of T‐cells capable of recognizing the pMHC complex.^[^ ^19^ ^]^ In this study, using pMHC thermal denaturation assays and pMHC comparative dockings, we identified promiscuous T‐cell peptides capable of binding multiple MHC molecules via shared anchoring residues. Additionally, after analyzing HLA supertypes based on peptide‐binding patterns and performing in vitro characterization, our findings confirm that while MHC binding is essential for T‐cell recognition, it is not alone sufficient to activate T‐cells.

## 2 Results

### 2.1 Identification and Characterization of Immunogenic B‐Cell Epitopes

**Figure 1:**
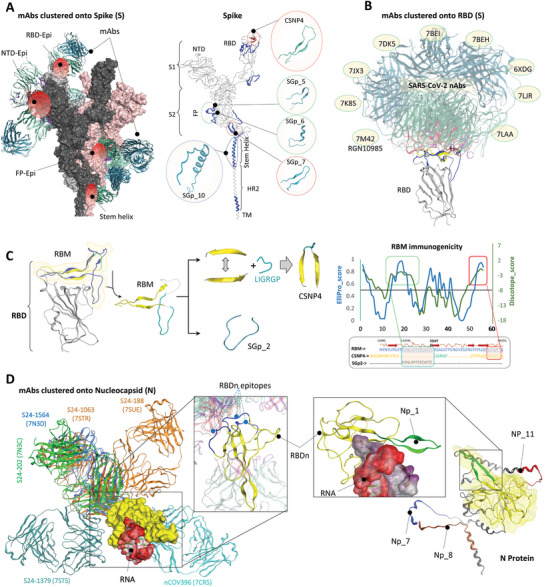
Monoclonal antibodies (mAbs) clustering‐based B‐cell epitope identification. A) Clustering of mAbs around Spike protein with experimentally confirmed epitopes (*left*). Epitopes are annotated on different domains of the Spike protein (*right*). The start and end positions of different domains, enzymatically active motifs, and other motifs of the Spike protein are shown (*bottom*). B) Clustering of FDA‐approved and other clinically active SARS‐CoV‐2 neutralizing mAbs around the RBD. The identifiers displayed in figure correspond to their RCSB PDB IDs. C) Selection of B‐cell epitopes based on the RBM motif. Based on ElliPro and Discotope, SGp2 was more immunogenic (*right*). D) Clustering of mAbs around RNA‐binding domain of nucleocapsid (RBDn) with experimentally confirmed epitopes (*left*). Selection of B‐cell epitope around entire Nuclocapsid protein using on ElliPro and Discotope (*right*).

To identify effective and structurally stable B‐cell epitopes, we implemented a specialized epitope‐paratope interface‐based (EPIB) selection strategy. This approach systematically explores immunogenic residues that are dispersed yet partially overlapped with neighboring epitopes, thereby enhancing the identification of potent epitopes. A crucial aspect of this method involves the clustering of experimentally confirmed nAbs around these overlapping epitopes. By leveraging extensive structural data, we identified functionally and immunologically active epitopes within the N‐terminal domain (NTD), RBD, and S2 domains of Spike, as well as the RNA‐binding domain of the Nucleocapsid (N) protein (RBDn) (**Figure** **1**A–E). Our approach, including EPIB, Discotope, and Ellipro screening, and conservancy filtration within the sarbecovirus family, identified ten epitopes within the Spike and eight epitopes within the N protein, some of which exhibited partial or complete overlap (Table S1, Supporting Information). Additionally, we selected seven peptides from ORF1ab and Membrane (M) proteins using Discotope and Ellipro screening methods.

Of the ten peptides within Spike, three were annotated in RBD, two around FP, and two in SH domains (Figure 1A). Notably, the peptides in the FP and SH regions partially overlapped with previously reported short and linear epitopes, specifically FP: PSKRSFIEDLLFNK and SH epitope: FKEELDKYF. However, predictions from Discotope and Ellipro identified highly immunogenic residues outside the core peptide regions, which were further validated by serum‐reactivity of those epitopes (discussed further, below). To date, receptor binding motif (RBM) has shown the highest efficacy in neutralization as exemplified by dozens of SARS‐CoV‐2 neutralizing FDA‐approved antibodies (Figure 1B). However, unlike linear FP and SH peptides, the spatial distribution of immunogenic residues in RBM is crucial to its immunodominance. Antibody–antigen clustering suggests that one of the two beta sheets in RBM is the most abundantly binding epitope; however, none of the previous studies have reported this motif as a linear epitope, likely due to its unstable folding.^[^ ^20^ ^]^ Considering this, we combined two structurally adjacent motifs through a linker peptide (LIGRGP) to form a structurally stable epitope (CSNP4, Figure 1C). Knowing that CSNP4 can potentially bind the Spike and block the RBD upward and downward movement, we have previously evaluated its SARS‐CoV‐2 pan‐variant inhibitory activity. Additionally, a flexible loop within the RBM was identified as a potential linear epitope, SGp_2, based on combined immunogenicity scores from Discotope and Ellipro (Figure 1C). Furthermore, a structurally resilient and highly conserved epitope SGp_7 was identified in the S2 domain (Figure 1A, *right*). Notably, the Moderna mRNA vaccine has been reported to induce antibodies targeting an epitope that overlaps with SGp_7,^[^ ^21^ ^]^ prompting its inclusion for further evaluation in our study.

EPIB screening around N protein identified a conformational epitope within the RBDn domain, which was subdivided into three highly flexible loops (Figure 1D, *middle*). Following multiple unsuccessful attempts to integrate the EPIB‐suggested immunogenic residues into a unified peptide scaffold, we opted for a conformational epitope termed “Np_1,” identified and recommended by both Discotope and Ellipro. Like CSNP4, where both beta sheets in RBM interact directly with ACE2, the two inverted beta sheets in Np_1 were found to directly interact with bound RNA (Figure 1D, right). In addition to this, seven other epitopes were selected in N protein, where Np_7 and Np_8 partially overlapped by “RQKKQQT” motif, located within the C‐terminus helix of the N protein (Figure 1E; Table S1, Supporting Information).

### 2.2 Selection of ID and IP B‐Cell Epitopes Using Human Sera

**Figure 2:**
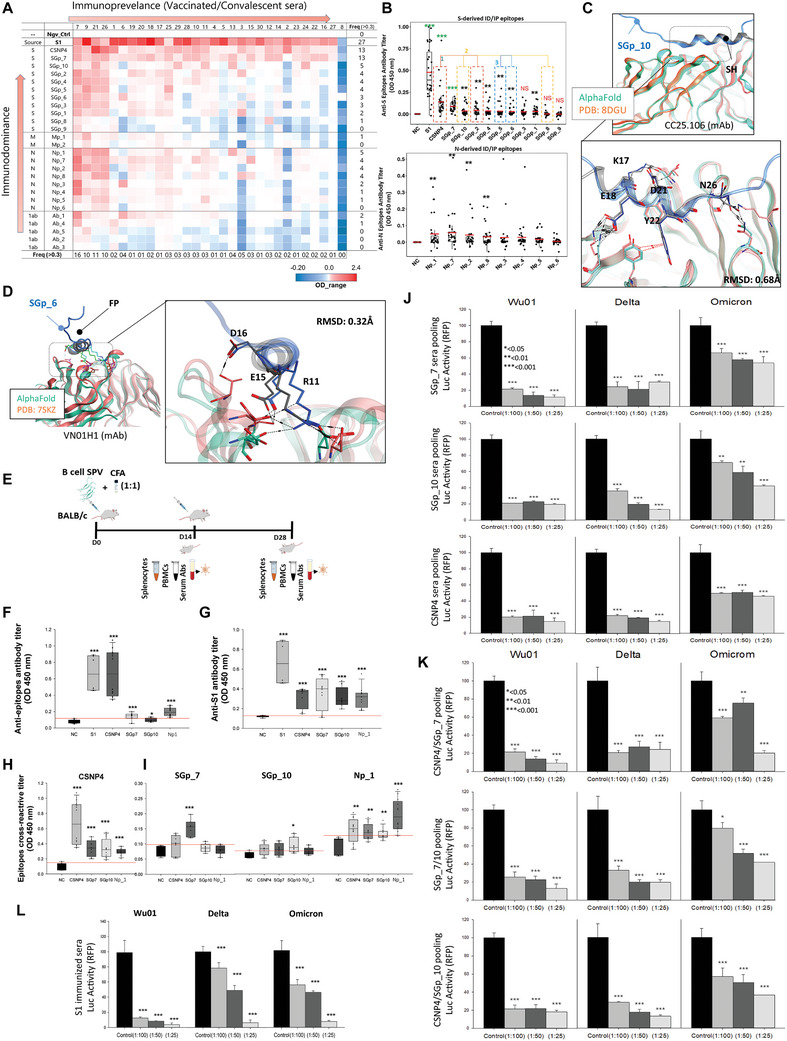
Immunodominant and Immunoprevalent B‐cell epitope selection and experimental evaluation. A) Anti‐peptide antibody titer in SARS‐CoV‐2 vaccinated individuals. The X‐axis displays the frequency of sera reacting with one peptide, while the Y‐axis indicates the strength of peptide reactivity with a single serum. B) Immunodominance of the Spike and Nucleocapsid derived peptides, based on Figure 2A,C) Superimposition of the AlphaFold 2 generated SGp_10/CC25.106 complex over the experimental structure to illustrate the availability of the stem helix for cross‐reactive immune response. D) Superimposition of the AlphaFold 2 generated SGp_6/VN01H1 complex over the experimental structure to demonstrate the availability of the fusion peptide for cross‐reactive immune response. E) Immunization regimen of the selected B‐cell epitopes in BALB/c mice. F‐I) Antibody titer in the immunized mice and their cross‐reactivity. F) Anti‐peptide antibody titers determined by reacting sera from mice immunized with S1, CSNP4, SGp_7, SGp_10, and Np_1. G) Anti‐S1 antibody titers of mice‐sera immunized with S1, CSNP4, SGp_7, SGp_10, and Np_1. H, I) Cross‐reactive antibodies from mice‐sera immunized with CSNP4, SGp_7, SGp_10, and Np_1. J) Antibodies from three pooled mouse sera immunized with the three peptides (SGp_7, SG_10, CSNP4) inhibit infection by all variants of concern (VOCs) of SARS‐CoV‐2 pseudovirus in hACE2‐293T cells. Luciferase reporter activity was measured in hACE2‐293T cells infected with Spike WU01, Delta, and Omicron pseudoviruses when treated with sera dilutions of 1:100, 1:50, and 1:25. Data are shown as mean ± standard deviation (compared with control versus *p < 0.05; **p < 0.01; ***p < 0.001; Student's *t*‐test). K) Combinations of antibodies from two mouse sera (CSNP4/SGp_7, SGp_7/SGp_10, CSNP4/SGp_10), immunized with the three peptides, inhibit infection by all variants of concern (VOCs) of SARS‐CoV‐2 pseudovirus in hACE2‐293T cells. Luciferase reporter activity was measured in hACE2‐293T cells infected with Spike WU01, Delta, and Omicron pseudoviruses when treated with sera dilutions of 1:100, 1:50, and 1:25. L) WU01, Delta, and Omicron Spike pseudovirus‐infected hACE2‐293T cells were treated with S1 antibodies from mouse sera immunized with the S1 protein at varying dilutions (1:100, 1:50, and 1:25), and their luciferase reporter activities were measured. Data are shown as mean ± standard deviation (compared with control versus *p < 0.05; **p < 0.01; ***p < 0.001; Student's *t*‐test).

To identify ID and IP B‐cell epitopes, we assessed 30 human sera from individuals who received two doses of COVID‐19 vaccines and were potentially exposed to SARS‐CoV‐2 infection. The presence of anti‐Spike antibodies in these samples was verified using ELISA (see methods). All 30 samples exceeded the medium control value and were classified as anti‐S seropositive (Figure S2A, Supporting Information). Sera #7 was found highly reactive while sera #8 was found inert in most of the reactions, including S1 protein as antigen (**Figure** **2**A). An epitope with OD values ≥0.3 and reacted with at least 3 sera, excluding #7 and #8, with the same intensity were considered as IP/ID.

The majority of anti‐N antibodies in the sera of recipients of the Pfizer and AstraZeneca vaccines are likely a result of natural COVID‐19 infections rather than the vaccinations themselves, as these vaccines do not specifically target the N protein.^[^ ^22^ ^]^ However, Sinopharm, an inactivated COVID‐19 vaccine, does induce profound anti‐N response.^[^ ^23^ ^]^ The human subjects in this study received mRNA COVID‐19 vaccines (first does AstraZeneca and second dose either AstraZeneca or BNT162b2), and the COVID‐19 infectivity was not disclosed at the time of blood samples collection. Thus, we investigated whether those sera that reacted with at least two peptides derived from non‐Spike antigens would be convalescent. A total of ten out of thirty samples were designated as convalescent based on the cutoff values. A cutoff value for the positive peptide‐sera reaction was established using the mean value plus three times the standard deviation of the negative control samples in each case, as described before.^[^ ^24^ ^]^ Due to the bulkiness and the fact that S1 subunit contains both NTD and RBD, it reacted with 27/30 sera. Six epitopes, particularly SGp_7, SGp_10, and CSNP4 were identified as ID/IP in Spike, and Np_1 and Np_7 in the N protein stood out as ID/IP candidates because they reacted with more than three sera (Figure 2A). No ID/IP candidates were identified in M‐ and ORF1ab‐derived epitopes (Figure S2B,C, Supporting Information). Based on overlap and domain sharing, we identified three pairs of Spike‐derived epitopes. Pair 1 includes CSNP4 and SGp_2, both situated within the RBM. Pair 2 consists of SGp_8 and SGp_10, which overlap at the SH peptide (Figure 2B). Pair 3 comprises SGp_5 and SGp_6, where SGp_6 includes the FP “PSKRSFIEDLLFN” that SGp_5 does not (Table S1, Supporting Information).

In pair 1, we found that CSNP4 reacted with 50% of the sera, supporting the EPIB‐based clustering of nAbs around this motif and its ID nature (Figures 1B and 2A).^[^ ^4^ ^]^ Notably, CSNP4, along with the S1 protein, was the most reactive ligand with sera (Figure 2B). In contrast, SGp_2 reacted with only three sera, despite being highly immunogenic according to Discotope and Ellipro. This discrepancy suggests that EPIB screening, combined with replacing the unstable P2 loop with the shorter “LIGRGP” linker, could provide a more effective approach compared to traditional B‐cell epitope design strategies.

In pair 2, SGp_8 and SGp_10 share an 11‐mer motif, “FKEELDKYFKN,” within the SH region, known for inducing broad‐spectrum neutralizing antibodies due to its conserved nature.^[^ ^9^ ^]^ A recent study on antibody responses in COVID‐19‐infected and vaccinated individuals reported a significantly higher antibody response to linear peptides derived from conserved areas of the S2 domain in infected individuals compared to vaccinated individuals.^[^ ^21^ ^]^ Given that our samples were primarily from vaccinated individuals, SGp_8, containing the conserved SH motif, did not exhibit reactivity with any sera (Figure 2A). However, SGp_10, a 38‐mer peptide with the SH motif in a helical structure, strongly reacted with more than three sera (OD > 0.3; Figure 2A,B). Subsequently, we assessed whether SGp_10 encompassed the antibody response elicited by SH using the AlphaFold multimer module. CC25.106, a mAb targeting this motif, exhibited a near‐perfect binding to the epitope residues in SGp_10 with minimal Root Mean Square Deviation (Figure 2C).^[^ ^9^ ^]^ This indicates that while the additional residues at the N‐ and C‐termini of SGp_10 enhance its immunogenicity, the core region retains its functional integrity.

In pair 3, SGp_5 strongly reacted with more than three sera, particularly sera #9, #21, #25, and #26, while SGp_6 showed moderate reactivity with two sera (see Figure 2A). Unlike SGp_10, which had additional residues flanking the SH motif in pair 2, SGp_6 in pair 3 contains the FP at its N‐terminus (Table S1, Supporting Information). This suggests that, like SGp_10, SGp_6 has the potential to induce broad‐spectrum neutralizing antibodies, as it shares paratopes with the VN01H1 mAb targeting the FP,^[^ ^25^ ^]^ as confirmed by the AlphaFold multimers modeling (Figure 2D).

### 2.3 The Spike‐Derived ID/IP B‐Cell Epitopes Elicit Robust In Vivo Antibody Response

Following the reaction response in human sera, we selected CSNP4, SGp_7, SGp_10, and Np_1 to evaluate their antibodies response in BALB/c mice. Mice received three doses of vaccines following standard vaccination regimen (illustrated in Figure 2E), and anti‐peptide or anti‐S1 antibody titers were subsequently analyzed. The S1 domain, containing the entire NTD and RBD domains of Spike, served as a control and elicited a strong anti‐S1 antibody response, consistent with a previous finding (Figure 2F).^[^ ^26^ ^]^ CSNP4 induced a significantly high anti‐CSNP4 antibody titer (*p* < 0.001) comparable to that of the S1 subunit, while other peptides induced relatively lower antibody titer in mice (Figure 2F).

Next, we cross‐analyzed the immunized sera for cross‐reactive antibody responses. We found that all four anti‐peptide sera reacted with the S1 (Figure 2G). Since CSNP4 is derived from RBD, CSNP4 elicits anti‐S1‐ or perhaps RBD‐specific immune response. However, the cross‐reactivity of SGp_7, SGp_10, and Np_1 antibody titer against S1 requires further confirmation. One possible explanation that we deduce was the partial overlap (3‐4 residues) of these peptide sequences with S1 protein. For example, Np_1 (ATRRIRGGDGKMKDLS) was partially overlapped with S1 at 402‐IRGDEVR‐408 motif (Figure S3A, Supporting Information), and SGp_10 (NNTVYDPLQPELDSFKEELDKYFKNHTSPDVDLGDISG) partially overlaps with S1 at 195‐KNIDGYFKIYS‐205. Additionally, the conformational flexibility of small peptides may expose epitopes that mimic native structural features of the S1 domain, further contributing to the observed cross‐reactive antibody response. Likewise, SGp_7 and SCp_10 and to some extent Np_1, sera revealed reactivity with CSNP4 (Figure 2H). However, the cross‐reactive antibody responses against SGp_7 and SGp_10 were minimal but specific to their own immune responses (Figure 2I). Np_1‐immunized sera also exhibited some level of cross reactivity with Spike‐derived peptides (Figure 2I). Taking together with these results, we suggest that while CSNP4 provides a robust immune response, SGp_7 response is potentially more effective against the mutated strains of the SARS‐CoV‐2, because SGp_7 is conserved not only among SARS‐CoV‐2 variants but also among other sarbecoviruses (Figure S3B, Supporting Information).

### 2.4 The Spike‐Derived ID/IP B‐Cell Epitopes Induce Broad‐Spectrum Neutralizing Antibodies Against SARS‐CoV‐2 Variants

To investigate whether the peptide vaccination induces SARS‐CoV‐2 neutralizing antibodies, we performed the pseudovirus neutralization assays as described previously.^[^ ^27^ ^]^ Sera from peptide‐immunized mice were tested against multiple SARS‐CoV‐2 variants, including Wuhan (Wu01), Alpha, Beta, Gamma, and Omicron (BA.1), under three conditions. 1) A single serum sample was assayed against all variants. 2) Pooled sera from three mice immunized with the same peptide was being assayed against all variants. 3) High‐titer sera from mice vaccinated with two different peptides were blended and assayed in a pseudovirus system. This strategy of serum pooling has been widely utilized in the past to tackle the immune escape of emerging variants and increase the breadth of neutralization of the convalescent or vaccinated human sera.^[^ ^28^, ^29^ ^]^ Under condition 1, SGp_7 sera inhibited the Wu01 variant (≈80% inhibition at three dilutions), Alpha (≈35% inhibition at 1:100 and 1:50 dilutions), and Delta variants (≈40% inhibition at 1:50 dilutions), but showed no response against other strains (Figure S4A, Supporting Information). Nonetheless, a profound neutralization (≈75% neutralization at all three dilutions) was achieved against Delta and Omicron variants with the SGp_7 sera, following condition 2 (Figure 2J). Likewise, SGp_10 could neutralize Wu01 (≈70% inhibition at three dilutions), Gamma (≈30% inhibition at 1:50 dilutions), and Delta (≈40% inhibition at 1:50 dilutions) variants (Figure S4B, Supporting Information). However, following condition 2 for SGp_10 sera, the Delta and Omicron variants were significantly neutralized in a serum‐dilution‐dependent manner (Figure 2J). CSNP4‐immunized sera neutralized all variants under condition 1. However, the neutralization response was more profound against Delta (≈85% inhibition at all dilutions) and Omicron (≈50% inhibition at 1:50 dilution) variants when condition 2 was applied (Figure 2J; Figure S4C, Supporting Information).

Next, we aimed to assess whether applying condition 3 would enhance neutralization efficacy and propose a potential multi‐epitope vaccination regimen for these vaccine candidates in future studies. Three different sera cocktails were formulated by blending CSNP4/SGp_7, SGp_7/SGp_10, and CSNP4/SGp_10 sera and assayed against Wu01, Delta, and Omicron variants at three different dilutions. We observed that compared to condition 2, the condition 3 regimen significantly neutralized SARS‐CoV‐2 immune‐escaped variants such as Delta and Omicron (Figure 2K). The Delta variant was neutralized by ≈80% across all three dilutions, while the Omicron showed more than 50% neutralization when CSNP4/SGp_7 sera were combined (Figure 2K, *top*). A consistent neutralization was observed when SGp_7/SGp_10 or CSNP4/SGp_10 condition was applied (Figure 2K, *middle and bottom*). These findings open a new opportunity for the cocktail (multi‐epitope) immunization of these candidates against emerging SARS‐CoV‐2 variants. Further studies are warranted to validate these promising results against live SARS‐CoV‐2 variants of concern (VOCs) in animal models immunized with the candidate peptides or a combination of multiple peptides. Such experiments, which require controlled biosafety facilities, are essential to confirm the clinical applicability and efficacy of these multi‐epitope strategies. In addition, we evaluated the pseudovirus neutralization by S1 immunized sera; however, due to the limited sera amount we could assay against Wu01, Delta, and Omicron variants only. More than 95% of the pseudo‐particles were neutralized at 1:25 dilution; however, the neutralization efficiency was reduced against Delta and Omicron variants at further dilutions (≈50% neutralization at 1:50 dilution) (Figure 2L). Taken together with these results, we suggest that the neutralization response is associated with the antibody titer within the immunized sera. CSNP4, which induced a high antibody titer comparable to S1 (Figure 2F), effectively neutralized the SARS‐CoV‐2 variants without pooling. However, the neutralization effect of SGp_7 and SGp_10 was less pronounced when treated as individual sera, likely due to the lower antibody titers induced by these peptides (Figure 2F). We also assumed that a relatively less conserved CSNP4 peptide could induce a higher antibody response compared to highly conserved SGp_7 and SGp_10 peptides.

### 2.5 Promiscuity of the HLA‐I Binding Peptide

The affinity of T‐cell peptides against their respective HLA haplotypes was evaluated by using differential scanning fluorimetry (DSF) analysis, where the Tm or the inflection temperature (Ti) indicates a thermal shift and pMHC dissociation/denaturation point. The affinity of the selected MHC‐I peptides was compared to the well‐characterized N protein‐derived HLA‐A*02:01‐restricted peptide “LLLDRLNQL”,^[^ ^30^ ^]^ with Ti of 75.5 °C recorded in our experiments. To identify high‐affinity promiscuous candidates, 27 peptides (Table S2, Supporting Information) were tested against the 14 HLA‐I types in 49 proposed combinations. Based on Ti values between 60 and 80 °C (classified as medium, good, or very good binding), 33 reliable pMHC pairs were identified (Figure 2A). However, no significant binding was observed for the 11 pMHC binders that were predicted as potential binders by the classical T‐cell epitope prediction strategy (Figure S2B, Supporting Information). Five pMHC pairs were highly unstable (Ti < 60 °C), while at least 10 candidates demonstrated equal or better MHC affinity compared to the control. Additionally, eight candidates exhibited binding to multiple HLA‐I alleles, indicating potential promiscuity (Figure 2A, *Left*).

**Figure 3:**
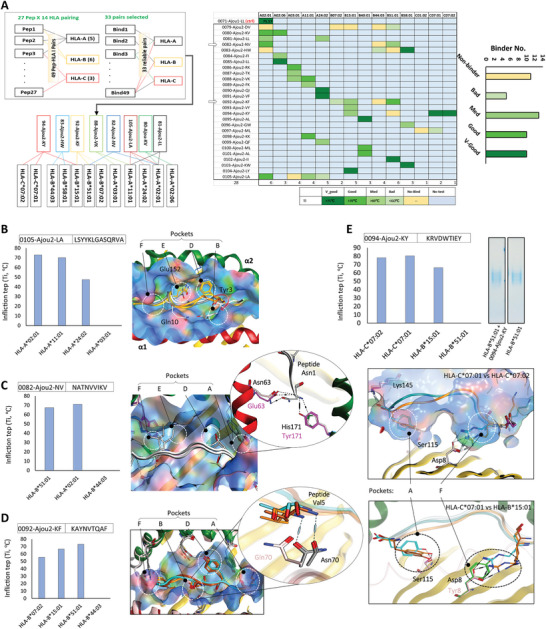
Promiscuous T‐cell epitope selection and evaluation. A) A simplified heatmap displaying the multi‐HLA binding of MHC‐I peptides. The heatmap is based on the inflection temperature of the pMHC complex, shown at the bottom. The number of bad, good, and v‐good binders is depicted in the bar plot (*right*). Promiscuous peptides are indicated in the left panel. B) The AlphaFold 2 generated pMHC complex of 0105‐Ajou2‐LA with A02:01 versus A11:01 (Left) and the DSF results of the same complexes (right). D) The AlphaFold 2 generated pMHC complex of 0082‐Ajou2‐NV with A02:01 versus B51:01 haplotypes (Left) and the DSF results of the same complexes (right). E) The AlphaFold 2 generated pMHC complex of 0092‐Ajou2‐KF with B51:01 versus B51:01 versus B07:02 haplotypes (Left) and the DSF results of the same complexes (right). Additionally, the AlphaFold 2 generated pMHC complex of 0094‐Ajou2‐KY with HLA‐C07:01 versus HLA‐C07:02 and HLA‐C07:01 versus HLA‐B*15:01 haplotypes (top) and the DSF results of the same complexes (bottom).

The promiscuity of MHC‐I‐binding peptides was analyzed by modeling their pMHC complexes with AlphaFold multimer to explore binding differences across haplotypes. MHCI contains a peptide‐binding cleft between the α1 and α2 helices of the α chain, crucial for anchoring residues of peptides.^[^ ^31^ ^]^ Peptide 0105‐Ajou2‐LA was assayed against four HLA‐A haplotypes in DSF. HLA‐A02:01 and HLA‐A11:01 showed the strongest affinity, while HLA‐A*03:01 did not bind (**Figure** **3**B, *left*). We found that the D pocket of these haplotypes possibly defines the peptide affinity as Tyr3 in “LSYYKLGASQRVA” fits in the D pocket of A*02:01 and A*11:01 but rotates by 180° in case of A*24:02 and A*03:01 (Figure 3B, *right*). The reliability of the AlphaFold proposed model was further validated by demonstrating the difference in E pocket between A*24:02 and A*03:01. HLA‐A*24:02 contains Glu152 in the α2 helix of E pocket that established a hydrogen bond with the Gln10 of “LSYYKLGASQRVA”. This is why A*24:02 showed a Ti = 47.5 °C whereas A*03:01 did not show any binding in DSF (Figure 3B, *left*). Peptide 0082‐Ajou2‐NV showed very good binding to A*02:01 and B*51:01 but failed to bind B*44:03. Here, we observed that the pocket A of these haplotypes played a pivotal role in peptide binding where the N‐terminal Asn1 of the peptide could interact with differing residues of the A*02:01 and B*51:01 (Figure 3C). Peptide 0092‐Ajou2‐KF showed promiscuity for haplotypes B*51:01 and B*15:01; however, B*07:02 was designated as bad‐binder (Ti = 55 °C). Interestingly, we found that B*07:02 differed within pocket B with B*51:01 and B*15:01, while most of the other pockets shared identity (Figure 3D). Both B*51:01 and B*15:01 contain Asn70 that interacted with the backbone nitrogen of Val5 of “KAYNVTQAF”; however, B*07:02 contains Gln70 that remained distant from Val5. The promiscuity of 0094‐Ajou2‐KY was seemingly defined by A and F pockets within C*07:01, C*07:02, and B*15:01. The Ti for C*07:01, C*07:02 was 80.4 and 78 °C, respectively, whereas for B*15:01 Ti was recorded as 66 °C (Figure 3F, *top*). While both C*07:01, C*07:02 shared identical pockets, particularly pocket F, B*15:01 had Tyr8 instead of Asp8 in the same pocket, which lost the strong salt bridges with Arg2 of the peptide “KRVDWTIEY” (Figure 3E, *bottom*). Although, computational screening suggested 0094‐Ajou2‐KY as potential B*51:01 binder; the DSF results showed only borderline binding. When validated through a native PAGE, we could not see any shift in the band, hence confirmed as non‐binder (Figure 3F, *top*).

### 2.6 HLA Supertypes and Peptides Anchor Points Investigation

**Figure 4:**
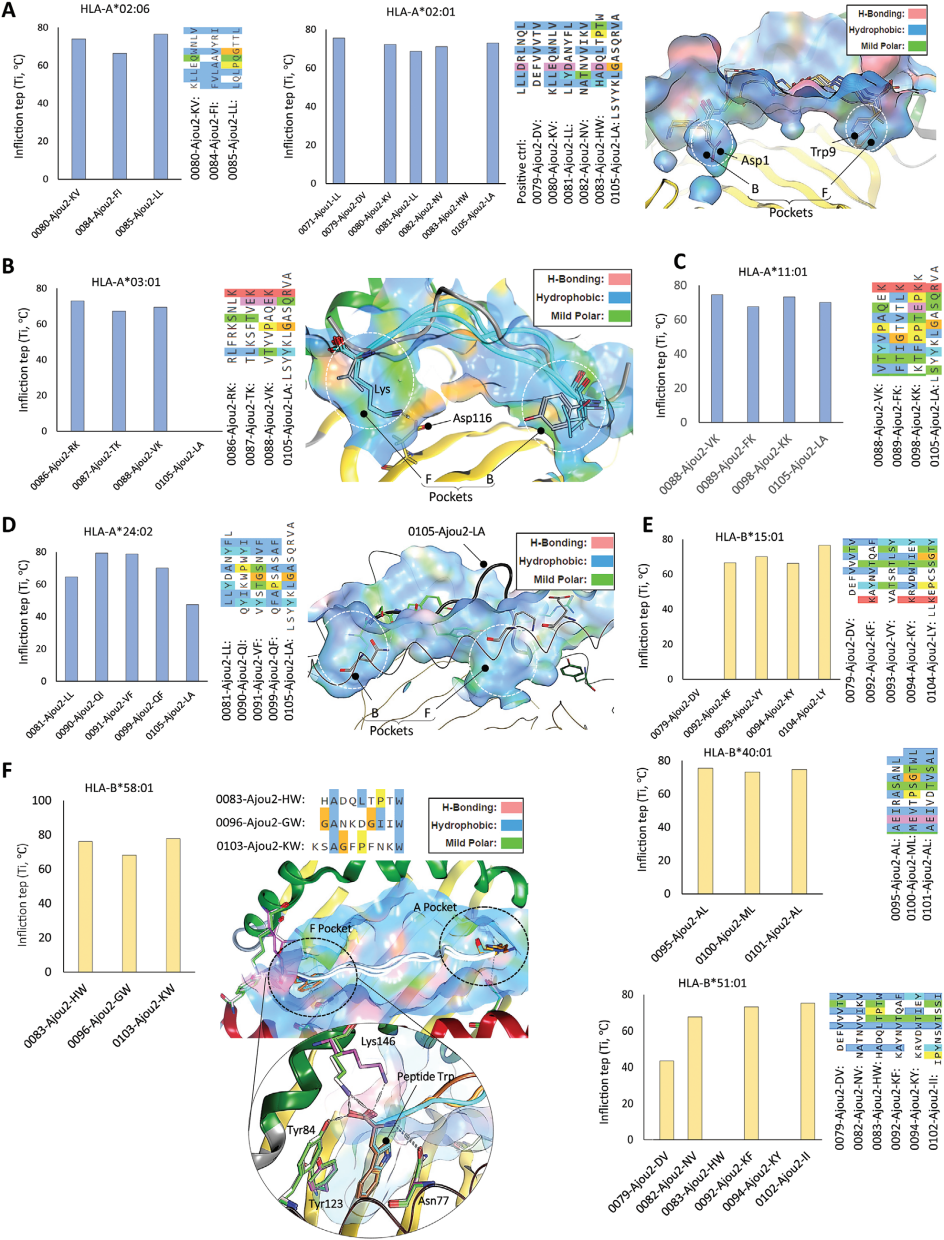
HLA‐I supertyping using multiple peptides and DSF and structural analysis. A) Bar plots based on inflection temperature showing the affinity of epitopes with HLA‐A02:06 and HLA‐A02:01. Peptides that did not show with binding HLA‐A*02:01 in DSF are shown in cartoon in comparison with control peptide. B) Bar plots based on inflection temperature showing the affinity of four epitopes with HLA‐A*03:01, while the AlphaFold 2 based model suggests differences in binding pockets. C) Bar plots based on inflection temperature showing the affinity of peptides with HLA‐A*11:01. D) HLA‐A24:02 affinity plots with multiple peptides. The right panel shows aligned peptide sequences and their anchor point similarities. 0105‐Ajou2‐LA deviates from anchoring and bulges out, shown in cartoon. E and F) HLA‐B binding affinity of peptides and their anchor residues' similarities.

HLA supertypes can influence the immune response by affecting the range of antigens recognized by T‐cells. Each supertype can be characterized by a super motif, representing the overarching main anchor motif recognized by HLA molecules within the respective supertype. For example, A2‐supertype molecules exhibit specificity for peptides with aliphatic hydrophobic residues at position 2 and the C‐terminus, while A3‐supertype molecules recognize peptides with small or aliphatic residues at position 2 and basic residues at the C‐terminus. Corroborating the importance of position 2 for A2 haplotypes, we found that A*02:01 and A*02:06 responded well to 9‐mer peptides containing Leu, Val, or Ala at position 2 (**Figure** **4**A). Additionally, the terminal residues in peptides with high affinity binding in DSF were major contributing anchors for both A*02:01 and A*02:06. However, in the case of the 0105‐Ajou2‐LA/A*02:01 complex, Ser2 remained away from anchoring, while Val12 contributed as a non‐canonical anchor residue. We further validated the position‐dependent anchoring of peptides on a structural basis by investigating A*02:01 non‐binding peptides 0079‐Ajou2‐DV and 9‐mer 0083‐Ajou2‐HW, and compared the results with the control peptide. We found that the B and F pockets of A*02:01 are predominantly hydrophobic which are best for fitting aliphatic hydrophobics residues (LIVMQ), but not acidic and bulky aromatics residues like Asp1 in 0079‐Ajou2‐DV and Trp9 in 0083‐Ajou2‐HW (Figure 4A, *right*).

The significance of the 2^nd^ and the last residues in a 9‐mer peptide was reaffirmed by DSF analysis of A*03:01 with four different peptides. As discussed above, peptides featuring small or aliphatic residues at position 2 and basic residues at the C‐terminus could identify the best binders against A*03:01.^[^ ^32^ ^]^ In contrast, 0105‐Ajou2‐LA, lacking these anchor motifs, failed to bind A*03:01 (Figure 4B). Upon closer examination, we found that the C‐terminal basic residues are crucial for binding to the F pocket of A*03:01, which contains the acidic Asp116 residue (Figure 4B, *right*). A*11:01 responded to all four tested peptides, which contain Ser and Thr at the second position and a basic residue at the C‐terminus (Figure 4C, *left*). This anchoring priority has been previously confirmed by other studies, where Thr and aliphatic hydrophobic residues (Val, Ile, and Leu) provide stronger anchoring at the second position, and Lys at C‐terminus.^[^ ^33^ ^]^ HLA‐A*24:02 prefers bulky aromatic residues at B and F pocket due to their deep hydrophobic cavities.^[^ ^34^ ^]^ We found that 0105‐Ajou2‐LA (13‐mer) does not contain any hydrophobic aromatics residues at the C‐terminus and exhibited a Ti of ≈47 °C and both the B and F pockets remained empty (Figure 4D, *right*). In addition, 081‐Ajou2‐LL a 9‐mer peptide contains Leu at second position but Phe8 near C‐terminus exhibited a Ti of 64. Three peptides, 090‐Ajou2‐QI, 091‐Ajou2‐VF, and 091‐Ajou2‐QF, which satisfied the anchoring criteria, exhibited the highest binding affinity (Figure 4D, *center*).

For HLA‐B haplotypes, previous studies have established the following anchoring preferences by peptides: B*15:01 prefers 9‐mer peptides with aromatic residues, particularly Phe or Tyr, at the C‐terminus.^[^ ^35^ ^]^ For B*40:01, two major anchoring points have been suggested: Glu or Asp at the second position and aliphatic amino acids at the C‐terminus.^[^ ^36^ ^]^ HLA B*51:01 prefers Ala, Gly, or Pro at the 2nd position and Phe, Ile, or Val at the C‐terminus.^[^ ^37^ ^]^ We found the exact anchoring match for B*40:01 in 0095‐Ajou2‐AL, 0100‐Ajou2‐ML, and 0101‐Ajou2‐AL, and for B*15:01 in 0092‐Ajou2‐KF, 0093‐Ajou2‐VY, 0094‐Ajou2‐KY, and 0104‐Ajou2‐LY (Figure 4E, *top panels*). For B*51:01, the anchoring match was identified in 0082‐Ajou2‐NV, 0092‐Ajou2‐KF, and 0102‐Ajou2‐II, whereas three peptides deviated from the anchoring criteria and therefore failed to show binding in DSF (Figure 4E, *bottom panel*). HLA‐B*58:01 predominantly prefers peptides with Trp or Phe at the C‐terminus and serine or Ala at the 2^nd^ position.^[^ ^38^ ^]^ We found that three peptides, i.e., 0083‐Ajou2‐HW, 0096‐Ajou2‐GW, and 0103‐Ajou2‐KW perfectly fit into its pocket and exhibited v‐good binding in DSF (Figure 4F). Taking these preferences into account, we observed that the peptides with the anchoring motifs bound preferentially to their respective HLA‐haplotypes with high affinity. Any variation in the anchoring residues either reduced the binding affinity or completely abolished the pMHC binding (Figure 4D,E). These findings suggest that the development of vaccines targeting common motifs present in relatively conserved epitopes recognized by T‐cells within specific HLA supertypes could potentially offer extensive coverage across diverse human populations.

### 2.7 Promiscuity of the HLA‐II Biding Peptides

Considering the in silico proposed promiscuity with multiple HLA‐DRB1 haplotypes and in some cases HLA‐DQ (refer to the multi‐HLA affinity step in Figure S1B, Supporting Information), five out of eight peptides were chosen from MHC‐II peptides for the IFN‐γ assay (Table S3, Supporting Information). To determine their potential promiscuity, we analyzed the variation in HLA‐DRB1 haplotypes (Figure S5A, Supporting Information). Box 1 in Figure S5A (Supporting Information), which encompasses peptides‐binding pockets 6, 7, and 9, is predominantly conserved in DRB1*03:01, DRB1*13:01, and DRB1*13:02 haplotypes; however, DRB1*03:01 exhibits variation in box 2, which also contributes to these pockets. Other HLA‐DRB1 haplotypes, including *01:03, *04:05, *09:01, *07:01, and *15:01, showed varying degrees of diversity in these pockets. Nevertheless, Leu67, Glu68, and Arg71 are conserved across all haplotypes in box 2.

**Figure 5:**
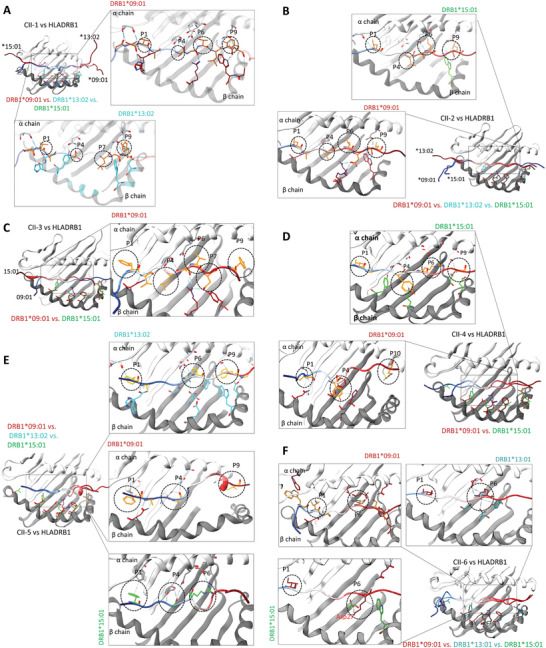
HLA‐II binding affinity and promiscuity as modeled through AlphaFold 2. A) Promiscuity of the CII‐1 peptide with DRB109:01 versus DRB113:02 versus DRB115:01. The comparison between DRB109:01 versus DRB113:02 shows favorable binding through pockets (P) 1, 4, and 9 (Right panels). B) CII‐2 exhibits favorable binding to DRB109:01 and DRB115:01 through P1, P4, P6, and P9. Aliphatic leucine serves as the main anchoring residue in CII‐2. C) DRB109:01 shows favorable binding with CII‐3 compared to DRB115:01. D) Both DRB109:01 and DRB115:01 favorably bind the CII‐4 epitope through C‐terminal basic residues. E) CII‐5 binds all three haplotypes (DRB109:01 versus DRB113:02 versus DRB115:01) mainly through the P6 pocket. F) CII‐6 utilizes Arginine 12 as the main anchoring residue to engage with the acidic residues of P6 of DRB109:01, DRB113:01, and DRB1*15:01.

Among MHC‐II peptides, CII‐1 was modeled with DRB1*09:01, DRB1*13:02, and DRB1*15:01 using the AlphaFold multimer and superimposed to emphasize the pocket residues and potential anchoring. Generally, peptides bind to the HLA‐II pocket from the N‐ to C‐terminal, with N‐terminal residues anchoring in Pocket 1 (P1) and so forth.^[^ ^39^ ^]^ However, we observed that the CII‐1 peptide did not adhere to this pattern with DRB1*15:01, instead anchoring at four points with DRB1*09:01 and DRB1*13:02 (**Figure** **5**A). In the case of the CII‐2 peptide, DRB1*13:02 was disregarded as a peptide binder, while DRB1*09:01 and DRB1*15:01 were engaged by the Leucine residues at P1, P4, P6, and P9 (Figure 5B). CII‐3 exhibited binding with DRB1*09:01, while CII‐4 was found to potentially bind to DRB1*09:01 and DRB1*15:01 (Figure 5C,D). CII‐5 and CII‐6, containing Arg at positions 7 and 12, respectively, were found to bind to DRB1*09:01, DRB1*13:01, DRB1*13:02, and DRB1*15:01 through the P6, which contains Asp27 and Glu10 (Figure 5E,F). Among HLA‐DQ, we evaluated the binding of CII‐1 and CII‐2 against QA101:02/QB105:01, and CII‐1 and CII‐4 against QA105:01/QB103:01. CII‐1 showed binding with QA105:01/QB103:01, while CII‐2 binds to QA101:02/QB105:01. Overall, these models suggest the multiple HLA binding affinity of all five peptides. However, additional experimental validation, such as Neoscreen, is needed to confirm and strengthen these findings.

### 2.8 IFN‐γ Induction by the T‐Cell Peptide in Human PBMCs

Upon activation, T‐cells undergo clonal expansion and differentiation, leading to the generation of effector T‐cells, including IFN‐γ‐secreting T‐cells. The production of IFN‐γ by T‐cells can be assessed experimentally using ELISA, intracellular cytokine staining followed by flow cytometry, or ELISPOT assays.^[^ ^40^ ^]^ Due to the low cell counts and failure in cell expansion in most of the limited‐sized hPBMCs samples, we were able to select only three PBMC samples that were sufficient for performing IFN‐γ assays for five of the MHC‐II peptides and nine MHC‐I peptides. Based on B‐cell peptide ELISA results, we found that samples #3, #4, and #29 strongly reacted with S1 but exhibited moderate reactivity with S‐ and N‐derived B‐cell peptides (Figure 2A). Nonetheless, we anticipated that the T‐cell peptides would activate T‐cells in the hPBMCs isolated from these samples. The hPBMCs were expanded by stimulation with CD3 and CD28 antibodies for 9 days to boot the T‐cell numbers, as described before.^[^ ^41^ ^]^ To prevent the blunting of sensitivity and cellular senescence resulting from T‐cell tolerance to stimuli, we allowed a maximum of 14 days between the commencement of expansion and evaluation of immunoreactivity (Figure S6A, Supporting Information). The number of cells and rates of spheroid formation increased steeply starting on day 4 of expansion, and by day 9, each sample underwent a cellular amplification of 20–100‐fold.

**Figure 6:**
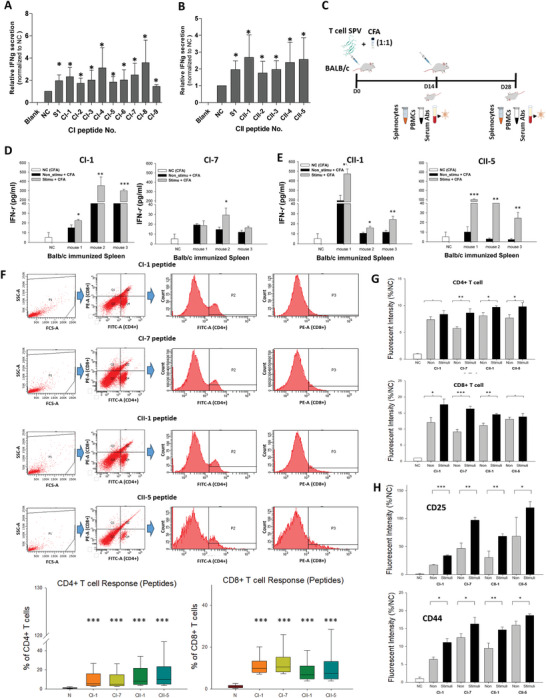
T‐cell Activation and Immune Response Profiling in Mice Immunized with MHC‐I and MHC‐II Peptide Candidates. A,B) S1 protein and peptide candidates, 9 for HLA class I & 5 for HLA class II were treated with expanded PBMC for 48 hrs. IFN‐γ was measured to confirm the T‐cell response. *P < 0.05, Kruskal‐Wallis multiple comparisons test was used to assess the positive value. C) Schematic representation of the immunization protocol. Mice (*n* = 3 mice per group) were vaccinated with CI‐1, CI‐7, CII‐1, and CII‐5 every 2 weeks. The IFN‐γ secretion by T lymphocytes of D) MHC‐I peptides and E) MHC‐II peptides from the splenocytes was detected using an IFN‐γ ELISA detection kit. F) Splenocytes were stimulated with T‐cell peptides and analyzed for surface marker expression using flow cytometry. Gates for CD4+ (FITC‐positive) and CD8+ (PE‐positive) cells were set based on single‐stained controls to determine the fluorescence positivity thresholds for each marker. A compensation matrix was applied to correct for spectral overlap between FITC and PE signals, using single‐stained controls for CD4‐FITC and CD8‐PE. Box plots, corresponding to FACS results, indicate the percentage of T cells induced by the T‐cell peptides, compared to control (CFA only). G,H) Splenocytes were stimulated with peptides to evaluate T lymphocyte isotype profiles. Bar plots illustrate the expression levels of surface markers CD4, CD8, CD25, and CD44 across different groups. Each group of splenocytes was stimulated with the peptide used for immunization and compared to a non‐stimulated (Non) and a control (NC (CFA only)) group. Normalization was performed against splenocytes immunized with PBS (CFA only). Data are shown as mean ± STD (compared with control versus *p < 0.05; **p < 0.01; ***p < 0.001; Student's *t*‐test).

Using S1 as a reference, we compared IFN‐γ levels induced by selected MHC‐I and MHC‐II peptides. Among the MHC‐I peptides, CI‐1, CI‐3, CI‐4, and CI‐6 were S‐derived; CI‐8 and CI‐9 originated from ORF1ab; CI‐5 from ORF7a; and CI‐2 and CI‐7 from the N protein (Table S3, Supporting Information). All MHC‐I peptides induced IFN‐γ in all three samples at levels comparable to or exceeding S1, with CI‐1, CI‐7, and CI‐8 consistently inducing the highest levels (**Figure** **6**A; Figure S6B, Supporting Information). For MHC‐II peptides, CII‐1 and CII‐5 consistently triggered IFN‐γ in all three PBMC samples (Figure 6B). Notably, in two out of three samples, CI‐1, CI‐7, CI‐8, CII‐1, and CII‐5 surpassed the IFN‐γ levels induced by S1.

### 2.9 Immunization in BALB/c Mice: Cross‐Reactivity and IFN‐γ Release

Our DSF and AlphaFold multimer modeling suggest that CI‐1 binds HLA‐A*02:01 and HLA‐B*51:01 and CI‐7 binds HLA‐B*15:01, and HLA‐B*51:01 as well as HLA‐B*07:02 at certain degree. Likewise, HLA‐II peptides CII‐1 and CII‐5 potentially bind more than two HLA‐DRB1 haplotypes (Figure 5A,E). Unfortunately, we were unable to recruit transgenic mice expressing these specific HLA combinations due to the inherent limitations of transgenic mouse models. Mice expressing multiple HLA alleles are uncommon, and generating such strains often requires sophisticated genetic engineering that may not align with study timelines. Instead, we evaluated four peptides in BALB/c mice for their T‐cell activation and IFN‐γ response. Although there are inherent differences between human and mouse MHC molecules, certain T‐cell epitopes are conserved across species and can be recognized by pan‐HLA molecules, such as PADRE (AKFVAAWTLKAAA), which is recognized by both human and murine MHC molecules and induces a strong T‐cell response.^[^ ^42^ ^]^

Before immunization, we predicted the murine MHC binding affinity of the four peptides against MHC‐I and MHC‐II molecules of mice, using SYFPEITHI, NetMHC‐IIpan 4.1, and NetMHCpan 4.1. We found that CI‐1 showed the best affinity with H2‐K^k^, both in NetMHCpan 4.1El and SYFPEITHI predictions. This peptide also showed binding with H2‐K^d^ in SYFPEITHI model (Table S4, Supporting Information). CI‐2 performed best against H2‐K^b^ in both prediction models (Table S4, Supporting Information). For class II murine MHC, a 15‐mer overlapping fragments of CII‐1 showed the best binding with H2‐E^k^ and H2‐A^d^. Likewise, CII‐5 performed best against H2‐D^b^ and a 10‐mer derivative of CII‐5 against H2‐E^d^ allele in SYFPEITHI scoring model; however, these alleles were not available on NetMHC‐IIpan 4.1 El interface.

To evaluate whether these peptides would induce T‐cell response in mice, we immunized BALB/c mice with these peptides for 4 weeks at 2‐week intervals (Figure 6C). The splenocytes of immunized and non‐immunized (CFA) mice were then stimulated with their respective peptides to evaluate the IFN‐γ response. Among the MHC‐I peptides, CI‐1 induced substantial cytokine response in all three splenocyte samples, compared to CI‐7 (Figure 5D). On the other hand, both MHC‐II peptides induced substantial IFN‐γ response in all three mice (Figure 6E). Splenocytes and PBMCs isolated from CFA‐treated mice were also activated with S1 and T‐cell peptides; however, none of the ligands elicited any IFN‐γ response in splenocytes or PBMCs (Figure S6C, Supporting Information).

### 2.10 Activation of CD4+ and CD8+ T Cells in BALB/c Mice by T‐Cell Peptides

It is well‐established that certain peptides, originally thought to bind exclusively to MHC‐I molecules, can also activate CD4+ T cells. This is attributed to the broader peptide‐binding pocket of MHC‐II molecules. For example, CTL peptides overlapping with those identified in our study were recently shown to activate both CD8+ and CD4+ T cells in SARS‐CoV‐2 and SARS‐CoV convalescent individuals.^[^ ^43^ ^]^ One such peptide, SARS‐CoV‐2‐N260‐270 (RTATKAYNV), which partially overlaps with CI‐7 (KAYNVTQAF) restricted to HLA‐B0702, HLA‐B1501, and HLA‐B5101, was found to activate both CD8+ and CD4+ T cells as confirmed via ELISPOT and FACS assays.^[^ ^43^ ^]^ Another study investigating memory T lymphocytes against SARS‐CoV‐2 in previously naive and previously infected individuals demonstrated that peptide M‐6 (QFAYANRNRFLYIIK) binds MHC‐II and activates CD4+ T cells, while its shorter form (YANRNRFLY) binds MHC‐I molecules.^[^ ^44^ ^]^ Notably, peptide M‐6 overlaps with CII‐5 (QFAYANRNRFLYIIK) in our study, which binds HLA‐DRB1*09:01, HLA‐DRB1*15:01, HLA‐DRB1*13:01, and HLA‐DRB1*13:02.

To validate the activation of CD4+ and CD8+ T cells by the identified T‐cell peptides, we employed FACS and ICC. FACS analysis revealed that splenocytes stimulated by both MHC‐I and MHC‐II peptides activated CD4+ and CD8+ T cells, with a stronger bias toward their respective T‐cell subsets. For instance, MHC‐II peptides induced a higher median percentage of CD4+ T cells compared to MHC‐I peptides, while MHC‐I peptides predominantly activated CD8+ T cells (Figure 6F). Additionally, FACS data corroborated IFN‐γ results, demonstrating that all T‐cell peptides elicited robust T‐cell responses compared to the CFA‐only control.

To further quantify T‐cell activation, we used ICC to measure fluorescence intensity. After multiple sampling and quantification, splenocytes isolated from T‐cell peptide‐immunized mice exhibited significantly higher CD4+ and CD8+ fluorescence intensity compared to CFA‐treated controls. Among MHC‐I peptides, CI‐7 induced robust activation of both CD4+ and CD8+ T cells, while CI‐1 was predominantly effective against CD8+ T cells. For MHC‐II peptides, CII‐5 demonstrated stronger activation of CD4+ T cells, whereas CII‐1 activated both CD4+ and CD8+ T cells (Figure 6g). Notably, the results related to CI‐7 are consistent with recent findings where the SARS‐CoV‐2‐N262‐270 peptide (RTATKAYNV), which overlaps with CI‐7, was demonstrated to induce IFN‐γ production in both CD4+ and CD8+ T cells, as confirmed by ELISPOT and FACS assays.^[^ ^43^ ^]^

To gain further insights into T‐cell activation, we assessed the expression of CD25 and CD44, two key markers of T‐cell activation and differentiation. CD25, the alpha chain of the IL‐2 receptor, is upregulated upon T‐cell activation to mediate IL‐2 signaling and proliferation.^[^ ^45^ ^]^ CD44, an adhesion molecule, is highly expressed on effector and memory T cells but remains at low levels on naïve T cells, serving as a hallmark of T‐cell activation and transition to a memory phenotype.^[^ ^46^ ^]^ In our experiments, splenocytes stimulated with both MHC‐I and MHC‐II peptides demonstrated significant upregulation of CD25 and CD44 markers compared to unstimulated cells and CFA‐only (Figure 6H; Figure S7, Supporting Information). These findings collectively demonstrate that the identified T‐cell peptides not only elicit robust CD4+ and CD8+ T‐cell responses but also promote the upregulation of key activation and memory markers, highlighting their potential to induce a comprehensive and durable cellular immune response.

Overall, these findings indicate that despite the absence of transgenic mice expressing the specific human HLA combinations, immunization of BALB/c mice with human HLA‐restricted peptides offers valuable insights into T‐cell activation and IFN‐γ response. Through predictive modeling and subsequent immunization, we demonstrate the potential cross‐reactivity and immunogenicity of these peptides in murine models, shedding light on their suitability for further investigation as potential sarbecovirus vaccine agents. However, it is important to note that the true protective efficacy of these peptides against SARS‐CoV‐2, particularly in the context of human infections, remains uncertain. Further studies, including infection challenge models and clinical validation, are warranted to fully assess their protective potential and translational relevance.

## 3 Discussion

This study proposes a systematic approach to identifying B‐cell epitopes from SARS‐CoV‐2 proteins, leveraging the available nAbs repertoire against Spike and N proteins, as well as human sera from vaccinated and COVID‐19 convalescent individuals. We employed cutting‐edge machine learning‐based epitope prediction tools within IEDB, alongside a novel EPIB‐based antibody clustering method and experimental validations, to design B‐cell‐immunogenic peptides that are conserved, functionally active, and structurally stable. By assessing their IP and ID in human sera, we identified epitopes located in conserved regions of SARS‐CoV‐2 proteins that elicited robust immune responses in mice. The anti‐peptides sera were shown to neutralize a wide range of SARS‐CoV‐2 pseudovirus variants, particularly when administered in cocktail formulations, which is akin to the strategy used in multi‐epitope vaccines.^[^ ^47^, ^48^ ^]^ This approach enhances neutralization effectiveness against immune‐escaped strains, providing a promising solution to address the evolving variants of SARS‐CoV‐2. In response to the inherent challenges in conducting in vivo studies, such as biosafety restrictions and limited access to authentic viral strains, we employed pseudovirus‐based assays. These assays, a reliable alternative, have been instrumental in screening peptide vaccines and evaluating nAb potency against SARS‐CoV‐2.^[^ ^24^, ^49^ ^]^

Beyond B‐cell epitopes, the promiscuity of T‐cell epitopes also plays a crucial role in vaccine design. The ability of peptides to bind multiple HLA haplotypes can significantly enhance immune coverage. Another aspect that profoundly reinforces the antigenicity of the T‐cell epitopes is their ID/IP potential. ID of the T‐cell peptides can be assessed by measuring the size of pHLA multimer‐positive T‐cell populations in responders, while IP is determined by the frequency of responders to each epitope within a cohort.^[^ ^50^ ^]^ Unfortunately, due to the limited availability of hPBMCs from all 30 human samples, we were unable to evaluate the ID/IP characteristics of the T‐cell peptides in this study. Alternatively, this study suggests promiscuous peptides binding to multiple HLA haplotypes, which is often influenced by key anchor residues interacting with specific peptide‐binding pockets. This supports the notion that structural compatibility is essential for optimizing peptide promiscuity.

Unlike conventional vaccines, which stimulate a broad immune response against multiple epitopes across an antigen, peptide‐based vaccines focus on specific antigenic regions to generate a more targeted immune response.^[^ ^51^ ^]^ This specificity is particularly beneficial when an immune response is required against a specific domain of a protein, such as mutated regions or neoepitopes in cancer‐associated antigens.^[^ ^11^ ^]^ Peptide vaccines are also effective in redirecting the immune response from variable regions of pathogen‐associated proteins to their conserved domains, improving their ability to target pathogens.^[^ ^10^ ^]^ This is particularly relevant in the context of COVID‐19, where a significant portion of the antibody response targets RBD, a highly variable domain, often compromising the efficacy of therapeutic antibodies and monovalent Spike‐based mRNA vaccines as the virus evolves into new VOCs.^[^ ^52^ ^]^ In addition, in terms of T‐cell response, Spike‐based mRNA vaccines predominantly stimulate Spike‐specific T‐cell responses, whereas the inactivated whole‐virus vaccines elicit a broader spectrum of T‐cell responses.^[^ ^53^ ^]^

To address these biased B‐ and T‐cell vaccine responses, multi‐epitope strategies like peptide cocktails and mRNA‐based vaccines have shown promise in clinical trials.^[^ ^10^, ^47^ ^]^ mRNA vaccines such as MIT‐T‐COVID encode multiple epitopes, generating strong T‐cell responses, and overcoming the limitations of monovalent mRNA vaccines.^[^ ^47^ ^]^ Another example, BNT162b4 (NCT05541861), includes epitopes from Spike, M, and ORF1ab proteins, inducing robust CD4+ and CD8+ T‐cell responses in animal models, reducing disease severity and viral titers.^[^ ^12^ ^]^ Peptide vaccine like CoVac‐1, which combines immunogenic peptides with toll‐like receptor agonist, XS15, as adjuvants, enhances broad and durable immune activation against SARS‐CoV‐2.^[^ ^10^, ^54^ ^]^ When evaluated in a clinical trial, CoVac‐1 was found safe and effective for up to 12 months (NCT04546841). It also induced stronger IFN‐γ responses in B‐cell‐deficient patients compared to mRNA vaccines and boosted pre‐existing T‐cell responses from prior mRNA vaccination. Importantly, it maintained efficacy against Omicron mutations.^[^ ^54^ ^]^ These findings highlight the potential of multi‐epitope vaccines in enhancing immune responses and combating diverse viral targets.

However, challenges remain, particularly regarding the limited availability of hPBMCs from the 30 human samples, which prevented comprehensive evaluation of the ID/IP characteristics of T‐cell peptides. Additionally, while the pseudovirus‐based assays provide valuable insights,^[^ ^24^, ^49^ ^]^ live virus challenges would provide more comprehensive data on immune efficacy in real‐world conditions. While we and others used CFA due to its availability and cost‐effectiveness,^[^ ^55^, ^56^, ^57^ ^]^ it is associated with confounding effects such as local inflammation and potential toxicity.^[^ ^58^ ^]^ Alternative adjuvants, such as toll‐like receptor ligands including Poly (ICLC), XS15, and CpG DNA, have demonstrated superior immunostimulatory effects and safety profiles, as exemplified by their use in CoVac‐1 and other peptide vaccine formulations.^[^ ^10^, ^54^ ^]^ Future studies should incorporate long‐term immunization protocols to assess memory responses and investigate the use of more potent and safe adjuvants to further enhance vaccine efficacy and safety profiles.

To address immune escape by SARS‐CoV‐2 variants, we employed a strategy of mixing sera from mice immunized with different B‐cell peptides. This approach showed promise in mitigating vaccine‐related complications such as vaccine‐induced myocarditis and viral infections diagnosis.^[^ ^59^, ^60^ ^]^ In addition, sera‐pooling has been widely used in controlling immune‐escaped SARS‐CoV‐2 VOCs.^[^ ^28^, ^29^ ^[^ Nonetheless, peptide cocktail or mRNA‐based peptide vaccination regimens may provide a better immune response.^[^ ^10^, ^54^ ^]^ Collectively, this study establishes a strong foundation for the development of multi‐epitope vaccine strategies targeting SARS‐CoV‐2 and other rapidly evolving pathogens, underscoring the critical role of innovative approaches in addressing current and future infectious disease challenges.

### 3.1 Limitations

For MHC‐II peptides structural analysis and peptide‐MHC binding predictions, we relied on the predictive modeling techniques such as AlphaFold. While these methods offer valuable insights, they are based on machine learning techniques and may not entirely capture the complexity of peptide‐HLA interactions in vivo. Experimental validation of these predictions using assays like NeoScreen^[^ ^61^ ^]^ would have further benefited the study. in addition, the assessment of immunogenicity in murine models, particularly in BALB/c mice, may not fully represent the human immune response. Differences in MHC molecules between humans and mice can affect the binding affinity and immunogenicity of peptides. Utilizing transgenic mice expressing human HLA alleles could provide more relevant insights.

## 4 Experimental Section

### B‐Cells Immunogenic Epitopes Screening

The full‐length structures of SARS‐CoV‐2 protein were either retrieved from RCSB PDB and AlphaFold databases or constructed using AlphaFold monomer module, if not available. Around 300 S‐binding and SARS‐CoV‐2 neutralizing antibodies were selected from SAbDab and CoV‐AbDab databases and downloaded from RCSB PDB. In case of structure unavailability, their 3D models were built by MOE 2022.2 or the AlphaFold multimer module. Likewise, the N and M binding antibodies were collected for epitope analyses. A two‐step strategy was used to identify conserved and immunogenic B‐cell epitopes for the SARS‐CoV‐2 structural and accessory proteins (Figure S1A, Supporting Information). First, the epitopes of structural proteins including Spike, N, and M were identified by clustering the available antibodies onto their respective antigens using EPIB strategy (discussed in results). For this, structural superimposition, AlphaFold multimers modeling, and MOE epitope–paratope identification modules were implemented.^[^ ^62^ ^]^ Detailed antibody‐antigen docking and epitope identification were described previously.^[^ ^20^ ^]^ Second, the B‐cell epitopes of all antigens (structural and non‐structural) were identified using Discotope, Ellipro, and confirmed with that of IEDB resource.^[^ ^63^ ^]^ Common or overlapping epitopes identified by both methods were selected for the shared antigens (Spike, N, and M), while conserved epitopes for non‐shared antigens were selected by the second strategy. In the case of short and linear epitopes, where the structural folding appeared unstable, adjacent secondary structures were included to stabilize their folding, after epitope annotations within the full‐length proteins.

### HLA type selection

Fourteen HLA‐I alleles, comprising five HLA‐A, six HLA‐B, and three HLA‐C variants, were chosen based on their expression and frequency in the Korean population, as determined in a study involving 5802 individuals.^[^ ^64^ ^]^ The alleles were expressed as the following percentage frequencies: A*02:01 (16%), A*02:06 (10.2%), A*03:01 (1.6%), A*11:01 (10%), A*24:02 (20.4%), B*07:02 (3%), B*15:01 (9.2%), B*40:01 (3.7%), B*44:03 (10%), B*51:01 (9.4%), B*58:01 (7%), C*01:02 (17%), C*07:01 (3.2%), and C*07:02 (7.8%).^[^ ^64^ ^]^ HLA‐DRB1 haplotypes were selected based on a separate study, with the following frequencies: DRB101:01 (5.9%), DRB101:03 (2.1%), DRB103:01 (2.7%), DRB104:05 (8.2%), DRB107:01 (6%), DRB109:01 (10.2%), DRB111:01 (5.5%), DRB113:01 (2.7%), DRB113:02 (7.4%), and DRB115:01 (7%).^[^ ^65^ ^]^ Furthermore, HLA‐DQ and HLA‐DP alpha/beta pairs were selected based on their prevalence in previous studies,^[^ ^66^ ^]^ including HLA‐DQA101:02 (17%)/HLA‐DQB105:01 (8.5%), HLA‐DQA105:01 (3%)/HLA‐DQB103:01 (14.3%), HLA‐DPA101:03 (44%)/HLA‐DPB102:01 (25%), and HLA‐DPA101:03 (44%)/HLA‐DPB104:01 (7%). Haplotypes with a frequency of less than 5% in the Korean population were also considered due to their high prevalence worldwide, as documented in the Allele Frequency Net Database.^[^ ^67^ ^]^

### MHC‐I and MHC‐II Binding Peptides Selection

For MHC‐I, 9‐13‐mer peptides were identified in structural and non‐structural proteins of SARS‐CoV‐2 using NetMHCpan EL 4.1, NetMHCCon, and the IEDB consensus methods.^[^ ^63^ ^]^ Shared or overlapping epitopes by these modules were selected and further sorted based on ANN 4.0 and SMM score, with IC50 < 500 nm pMHC binding affinity. For MHC‐II binders, relatively longer ligands (14–22‐mer peptide) were screened, using NetMHC‐IIpan EL 4.3,^[^ ^68^ ^]^ and IEDB consensus 2.22 methods. Ligands with IC50 < 500 nm affinity and best‐combined scoring of NN‐align IC50, SMM‐align IC50, and NetMHC‐IIpan IC50 were selected.^[^ ^69^ ^]^ The stepwise procedure is outlined in Figure S1B (Supporting Information). Next, the candidates were virtually screened, using an induced‐fit‐docking module with MOE suite against multiple HLA to identify high‐affinity promiscuous epitopes for both HLA‐I and HLA‐II haplotypes. The candidate peptides with high HLA‐affinity (based on docking scores) were modeled using AlphaFold multimers module. Finally, poor‐quality peptides were filtered out by applying seven‐point final selection criteria (Figure S1B, Supporting Information).

### Peptides Synthesis and Characterization

The manufacturability of the peptide was evaluated using the method described previously.^[^ ^70^ ^]^ B‐cell and HLA‐II epitope peptides with no plights in manufacturability were synthesized at ≥90% purity (GenScript, Piscataway, NJ, USA). HLA‐I peptides were synthesized through GeneCust (Boynes, France) at ≥ 90% purity. The purity of HLA‐I peptides was confirmed by HPLC using ChromCore120 C18 Naco Chrom 5 µM, 4.6 × 250 mm column. For HLA‐II and B‐cell peptides purity confirmation, Inertsil ODS‐SP 4.6 × 250 mm column was used. The HPLC purity and MS reports of all peptides are provided in Supporting Information. For stock solution preparation, lyophilized peptides were suspended in dimethyl sulfoxide or distilled water at a working concentration of 10 mg/mL and stored at −20 °C.

### Peptide‐MHC Binding Assay (DSF)

The binding affinities between peptides and HLA‐I alleles were evaluated using DSF (Imusyn Hannover, Germany). Briefly, for measurements, synthetic peptides were added at a ten‐fold molar excess to HLA‐I molecules, which contained several endogenous peptides with unique melting temperatures (Tm). Peptide binding affinity was measured as the change in intrinsic protein fluorescence during a temperature ramp. A relatively steep temperature change of 3 °C min^−1^ was implemented during the measurement. Three types of samples were measured: HLA only, peptide only, and HLA plus peptide. For negative controls, measurement of HLA only, containing only endogenous peptides, was utilized. Peptides containing more than one tyrosine and/or tryptophan often do not yield clear DSF measurements. To overcome this, the peptide measurements were subtracted from the HLA plus peptide measurements. Otherwise, HLA‐peptide binding was confirmed by loading the complex onto a native PAGE and comparing the results to those of HLA‐native peptides PAGE results, as previously described.^[^ ^71^ ^]^

### Blood Sample Collection and Validation

Thirty blood samples were obtained from volunteers who had received two or more doses of the mRNA vaccines recommended by the World Health Organization (WHO), along with additional booster doses. However, their COVID‐19 convalescence was undisclosed at the time of sample collection. The Human SARS‐CoV‐2 Spike IgG ELISA Kit (Thermo Fisher Scientific, BMS2325, MA, USA) was utilized to confirm the presence of anti‐S antibodies in blood samples. However, this kit detects only anti‐S antibodies and does not confirm the COVID‐19 convalescence.

### Ethical Approvals for Animal and Human Studies

The blood samples were provided by the Ajou University Human Resource Bank (AHBB, Suwon), and were collected under the guidance of the Environmental Health and Biosafety Program from healthy adult volunteers who provided informed consent in accordance with IRB protocols. The Institutional Review Board (IRB) of Ajou University Hospital has approved this study (IRB No. AJOUIRB‐EX‐2023‐123).

### Anti‐Peptides Antibodies Titer Estimation (Peptides ELISA)

Ninety six‐well immuno‐plates (SPL Life Science, Korea) were coated with 10 µg mL^−1^ peptides and 100 ng mL^−1^ S1 protein (AcroBiosystems, S1N‐C52H3‐100UG, USA) as positive control per well, at 4 °C overnight. The S1 subunit of SARS‐CoV‐2 was previously reported to induce better anti‐SARS‐CoV‐2 immune response as a subunit vaccine, compared to RBD.^[^ ^26^ ^]^ After washing with 0.1 triton X‐100 in PBS, the plates were blocked with Protein‐Free Blocking Buffer (protein‐free compound in phosphate‐buffered saline, pH 7.4) at RT for 1 h and incubated with 100 µL of different dilutions of human or mouse serum at 37 °C for 2 h. Reacted serum was detected using HRP‐conjugated goat anti‐human IgG and chromogenic substrate TMB (ThermoFisher, Waltham, MA, USA). A cutoff value for the positive peptide‐sera reaction was established using the mean value plus three times the standard deviation of the negative control samples in each case.

### Human PBMCs Isolation, Expansion, and IFN‐γ Secretion Assay

After ID/IP investigation of B‐cell peptides (described later), ten human samples were considered convalescent and selected for hPBMCs isolation and subsequent IFN‐γ assays. Convalescent samples were prioritized because most T‐cell peptides identified in this study were non‐Spike‐based, necessitating hPBMCs that were exposed to SARS‐CoV‐2 proteins beyond the Spike protein. Due to the limited sample amounts, three out of ten samples could generate enough hPBMCs (4 × 10^6^–1.5 × 10^7^ cells) for T‐cells IFN‐γ assays. hPBMCs were isolated by Ficoll‐Paque (Amersham Biosciences, Uppsala, Sweden) gradient centrifugation, collected as non‐adherent cells, and cultured in complete medium (RPMI1640, supplemented with 10% FBS, 100 U mL^−1^ penicillin, 100 µg mL^−1^ streptomycin).

To facilitate T lymphocyte differentiation, hPBMCs were cultured in a 24‐well culture plate with 2 mL of complete medium. The plates were incubated in a humidified 37 °C environment with 5% CO₂ and cultured for 9–14 days in the presence of 1 µg mL^−1^ CD3/CD28 stimulating antibodies (catalog numbers 14‐0037‐82 and 14‐0289‐82, Thermo Fisher Scientific, Waltham, MA, USA) until cell expansion was observed. After the differentiation of the T lymphocyte, cells were seeded with a final working concentration of ≈7 × 10^5^ cells per well in 96 well plates with 0.1 mL complete medium for 24 h. The next day, peptides 1 µg per well and S1 protein 100 ng per well were treated with each sample to activate the cells and after 2 days, the supernatant from each well was utilized for ELISA to quantify IFN‐γ levels using the Human IFN‐gamma Quantikine ELISA Kit (R&D Systems, catalog number DIF50C, MN, USA). Although CD3 and CD28 activation induced a high background level of T‐cell activation, the inclusion of a no‐peptide‐treated control allowed to measure this background and distinguish peptide‐specific IFN‐γ responses. Additionally, S1 was used as a positive control to validate the functionality of the assay.

### Mice Immunization with B‐Cell and T‐Cell Peptides

Six‐weeks‐old female BALB/c mice (ORIENT BIO Inc, Korea) were raised under specific pathogen‐free standard conditions. Animals were housed in groups (*n* = 4 per group) at ambient temperature (18–24 °C) and 40–60% humidity, fed ad libitum with a 20% protein diet, and maintained on a 12‐h light/dark cycle. For B‐cell vaccines immunization, mice were grouped into PBS, S1 (subunit vaccine), and four B‐cell peptide groups (CSNP4, SGp_7, SGp_10, SGp_11) (Table S1, Supporting Information) and administered subcutaneously with B‐cell peptides (100 µg/peptide/mouse), S1 (50 µg/mouse) or PBS, emulsified in (1:1) with Freund's complete adjuvant (CFA) (F5881, Sigma‐Aldrich, USA), as described previously.^[^ ^56^, ^57^ ^]^ Each group received their first injection at day 0 and boosters at day 14 and 28. Blood samples were collected, 3 days after the first and last immunization, for peptide ELISA and SARS‐CoV‐2 Spike pseudovirus neutralization, discussed below under the section “SARS‐CoV‐2 pseudovirus neutralization assay”.

For T‐cell vaccine immunization, mice were administered T‐cell peptides (CI‐1, CI‐7, CII‐1, CII‐5) (Tables S2, and S3, Supporting Information) at 100 µg/peptide/mice, S1 (50 µg/mouse) or PBS, emulsified with CFA (F5881, Sigma–Aldrich, USA) at 2‐week intervals and sacrificed three days after second and third vaccination, following the same protocol as B‐cell peptides vaccination and as described before.^[^ ^56^ ^]^ 35 days after the booster dose is sufficient time to capture the peak of effector T‐cell activation, as T cells were highly activated and produced cytokines shortly after antigen exposure.^[^ ^72^, ^73^ ^]^ The splenocytes were isolated and gently homogenized using the top of a 10 mL syringe as a plunger and filtered through a cell strainer under sterile conditions. The splenocytes and PBMCs were separated using Ficoll‐Paque (Amersham Biosciences, Uppsala, Sweden) gradient centrifugation method as described earlier.^[^ ^74^ ^]^ The relative IFN‐γ levels in the splenocytes stimulated with T‐cell peptides were measured and compared to those from PBS (CFA)‐treated and immunized but non‐stimulated cells using the Mouse IFN‐gamma Quantikine ELISA Kit (R&D Systems, catalog number MIF00, MN, USA), following the manufacturer's protocol.

### SARS‐CoV‐2 Pseudovirus Neutralization Assay

SARS‐CoV‐2 Spike pseudovirus particles for each variant of concern, including Wuhan (Wu01), Beta, Gamma, Delta, and Omicron (BA.1) were generated as previously described.^[^ ^27^ ^]^ For the luciferase assay and mCherry fluorescence assay, hACE2‐293T cells (631289, Takara, USA) were seeded at a density of 8000 cells per well. The cells were infected with 40 µL of various pseudoviruses and subsequently treated with peptide‐immunized sera diluted at 1:100, 1:50, and 1:25 dilutions. The total reaction volume was 100 µL, and the plates were incubated in 37 °C CO₂ incubator for 48 h. The luciferase activity was measured according to ONE‐Glo Luciferase Assay System (Promega, USA, E6120) manufacturer's instructions, and a Synergy HTX Fluorometer (BioTek, USA) was used without attenuation. To evaluate the combination effects (synergy) of two different sera, neutralization against Wu01, Delta, and Omicron particles was demonstrated by mixing two sera in a (1:1) and treated at 1:100, 1:50, or 1:25 dilutions of CSNP4‐SGp_7, SGp_7‐SGp_10, and CSNP4‐SGp_10. For the mCherry fluorescence assay, hACE2‐293T cells were treated with pseudovirus in 100 µL media and incubated for 48 h. Images were then visualized using an Axiovert 200 fluorescence microscope (Carl Zeiss, Göttingen, Germany).

### Fluorescence‐Activated Cell Sorting (FACS) Analysis

The splenocytes were isolated from mice immunized with MHC‐I peptides (CI‐1, CI‐7), MHC‐II peptides (CII‐1, CII‐5), or CFA alone (control), as described earlier. The cells were washed with FACS buffer (PBS supplemented with 1% BSA) to remove any residual debris. Following isolation, splenocytes were treated with the peptides and then resuspended in 100 µL of one of the antibody panels listed below. Each panel contained a 1:50 dilution of the respective antibodies prepared in FACS buffer. For staining, cells were incubated with either FACS buffer alone (unstained control) or antibody mixtures at 4 °C for 1 h. After incubation, cells were washed thoroughly with FACS buffer to remove unbound antibodies and resuspended in 250 µL of FACS buffer for analysis. Flow cytometric analysis was performed using a FACSAria III flow cytometer (Becton Dickinson, NJ, USA). The antibodies used in FACS analysis are CD4‐FITC (Santa Cruz, sc‐19643 FITC, CA, USA), and CD8‐PE (Santa Cruz, sc‐70802 PE, CA, USA).

To ensure accurate identification of CD4+ and CD8+ T‐cell populations, the following gating strategy was employed: Splenocytes were first gated using forward scatter (FSC) and side scatter (SSC) to exclude cellular debris and select live, intact cells. Doublets and aggregates were excluded by plotting FSC‐A versus FSC‐H and gating single cells. Unstained controls were included to establish baseline fluorescence levels and identify autofluorescence for both FITC and PE channels. Gates for CD4+ (FITC‐positive) and CD8+ (PE‐positive) cells were set based on single‐stained controls to determine the fluorescence positivity thresholds for each marker. A compensation matrix was applied to correct for spectral overlap between FITC and PE signals, using single‐stained controls for CD4‐FITC and CD8‐PE. The compensation values were determined using single‐stained controls for FITC and PE. After gating and compensation, the percentage of CD4+ and CD8+ T cells was quantified from the gated populations.

### Immunocytochemistry (ICC) and Imaging

Splenocytes were fixed with 4% paraformaldehyde for 5 min at room temperature to preserve cellular structure. After fixation, the cells were permeabilized by incubation with phosphate‐buffered saline (PBS) containing 0.25% Triton X‐100 (Sigma–Aldrich) for 10 min at room temperature. Following permeabilization, the cells were washed three times with PBS to remove residual reagents and incubated in a blocking solution (PBS supplemented with 1% bovine serum albumin (BSA) and 0.1% Tween‐20) for 1 h at room temperature to minimize nonspecific binding. The cells were then incubated with the following primary antibodies: CD4‐FITC (1:100, Santa Cruz Biotechnology, sc‐19643 FITC, CA, USA); CD8‐PE (1:100, Santa Cruz Biotechnology, sc‐70802 PE, CA, USA); CD44‐FITC (1:100, Santa Cruz Biotechnology, sc‐53068 FITC, CA, USA); CD25‐PE (1:100, Thermo Fisher Scientific, 34577, NJ, USA). Antibody staining was carried out at 4 °C for 24 h to ensure optimal binding. After incubation, the cells were washed with PBS to remove unbound antibodies. The nuclei were counterstained using a DAPI‐containing mounting solution (Vector Laboratories, H‐1200, CA, USA). Stained cells were visualized under an LSM 710 Confocal Laser Scanning Microscope (Carl Zeiss, Göttingen, Germany), and images were acquired for analysis.

### Statistics and Reproducibility

All statistical analyses were conducted using SigmaPlot v12.5 (Systat Software, Inc., San Jose, CA, USA) and R package. Each experiment was performed in triplicate, and the results are presented as the mean ± standard deviation (SD). Statistical significance was assessed using a two‐tailed Student's *t*‐test, with a threshold of *p* < 0.05 considered statistically significant. The levels of significance are denoted as follows: *p* < 0.05 (*), *p* < 0.01 (**), *p* < 0.001 (***). Unless otherwise specified, all comparisons were made relative to the control group.

## Conflict of Interest

The authors declare no conflict of interest.

## Author Contributions

M.S., S.U.M, contributed equally to this work. M.S., S.U.M., and J.Y.S. performed experiments and data analysis. M.S., S.U.M., J.Y.S., J.H.C., and D.K. contributed to the writing of the manuscript, reviewed the results, and approved the final version of the manuscript. M.S. and H.G.W. conceptualized and designed the study, and performed data analyses. H.G.W. supervised and funded the study.

## Supporting information



Supporting Information

## Data Availability

The data that support the findings of this study are available in the supplementary material of this article.
