# Supplementary material for: Pan‐Variant SARS‐CoV‐2 Vaccines Induce Protective Immunity by Targeting Conserved Epitopes
Source: Adv Sci (Weinh). 2025 Feb 27;12(16):2409919. doi: 10.1002/advs.202409919 (PMC12021035; doi:10.1002/advs.202409919)

# **Supplementary data**

**Supplementary Figures**

**Figure S1. Overview of Antigenic Epitope Selection and Design Strategies for B Cells and T Cells**. A) Outline of B cell antigenic epitopes selection and design strategy. B) Outline of T cell epitope designing and selection strategy.


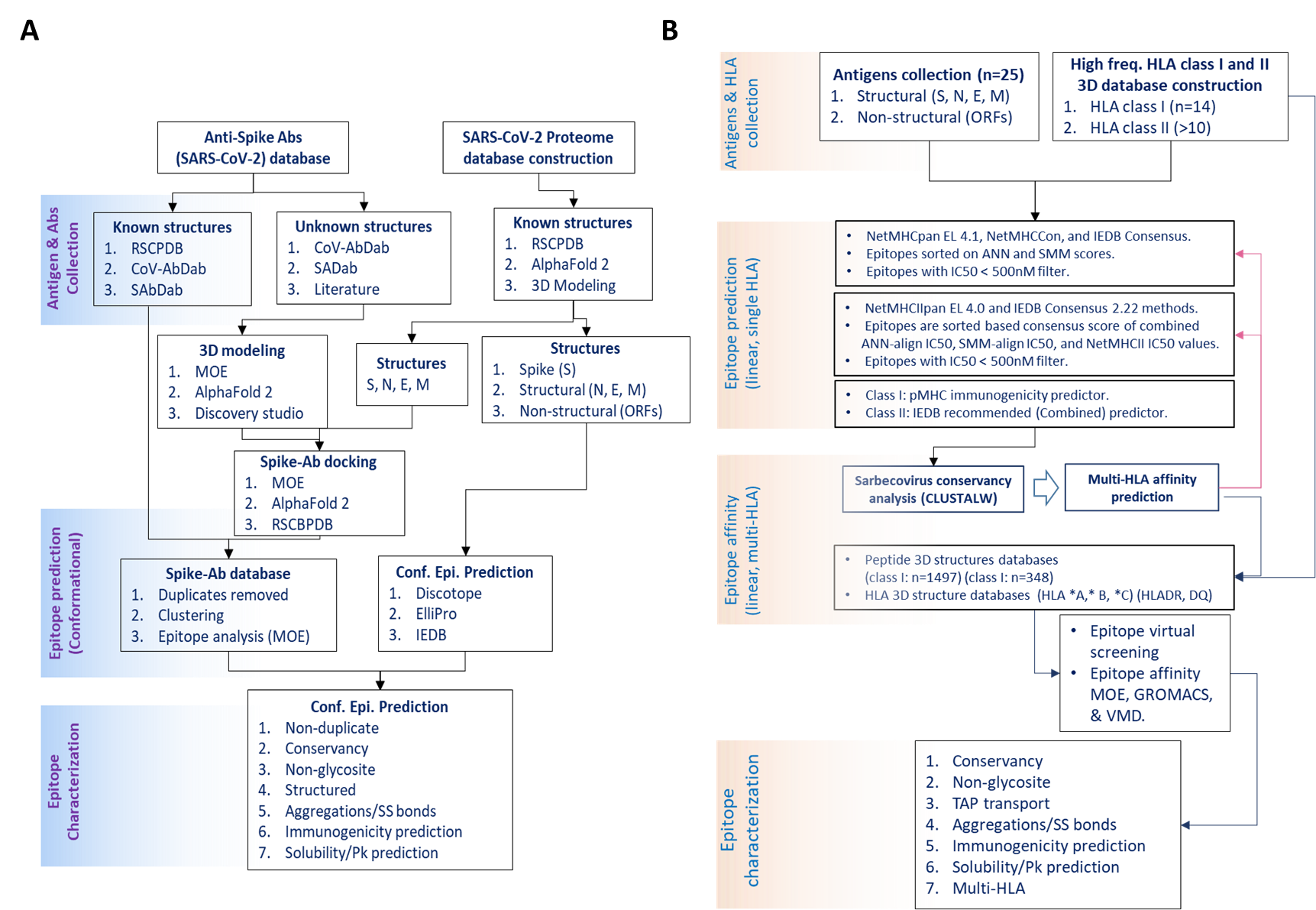


**Figure S2. Anti-peptides antibody titer evaluation withing SARS-CoV-2 vaccinated human sera.** A) Qualitative analysis of serum total IgG antibody against SARS-CoV-2 in healthy donors within 6 months post-vaccination determined by ELISA. Antibody retention in blood samples was determined by comparing them to a negative control and three positive controls (LP, MP, HP). Samples with OD values above the mid-control were considered to contain SARS-CoV-2 antibodies. Red line is cutoff values to determine positive samples. ***P < 0.001, Kruskal-Wallis multiple comparisons test was used to assess the positive value. B and C. Anti-peptide antibodies titer. A cutoff value for the positive peptide-sera reaction was established using the mean value plus three times the standard deviation of the negative control samples in each case.


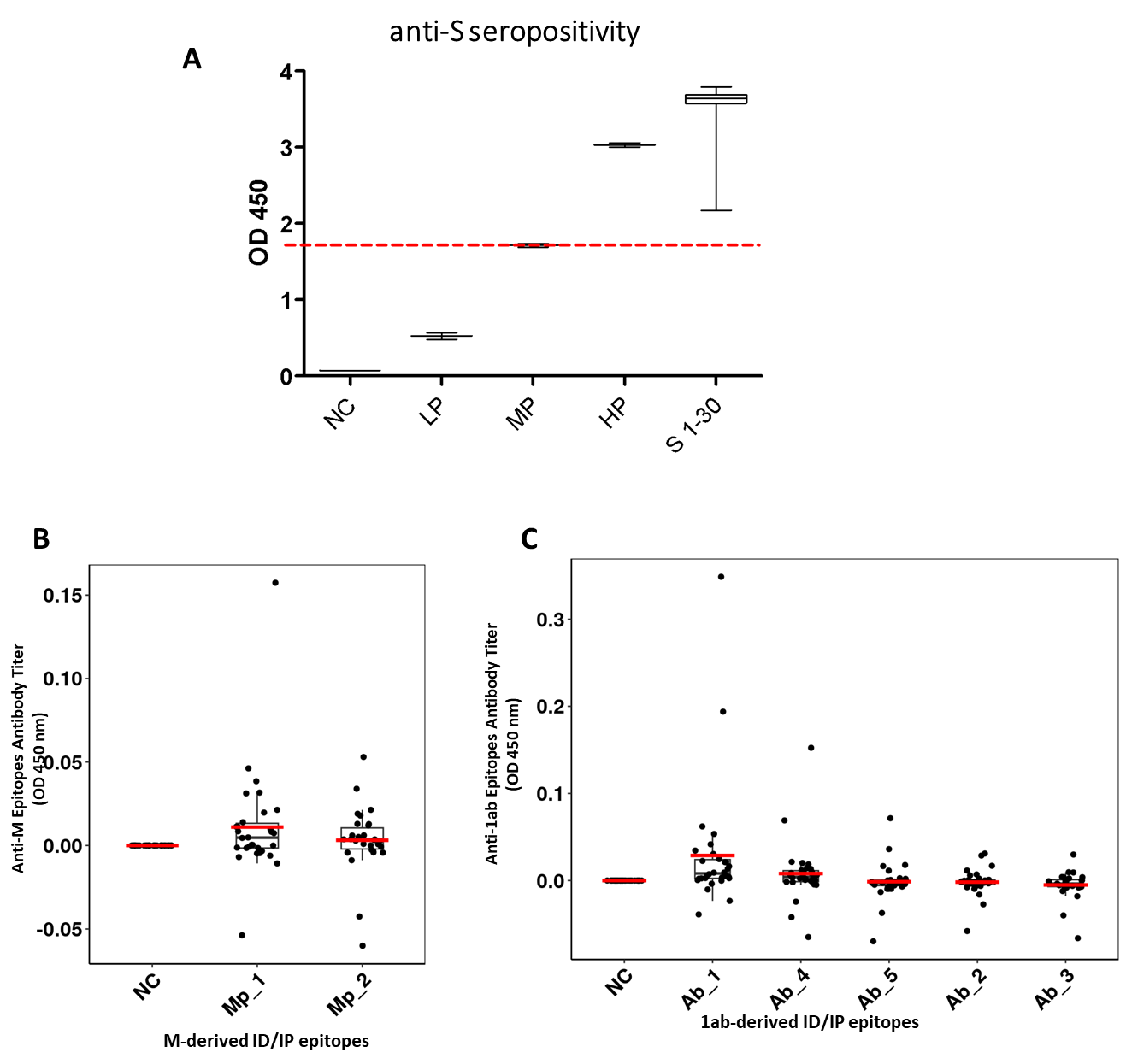


**Figure S3. (A) Similiarty between Np_1 peptide and S1 domain of Spike. (B) Multiple sequence alignment of the sarbecovirus Spike, Nucleocapsid and Membrane proteins.**


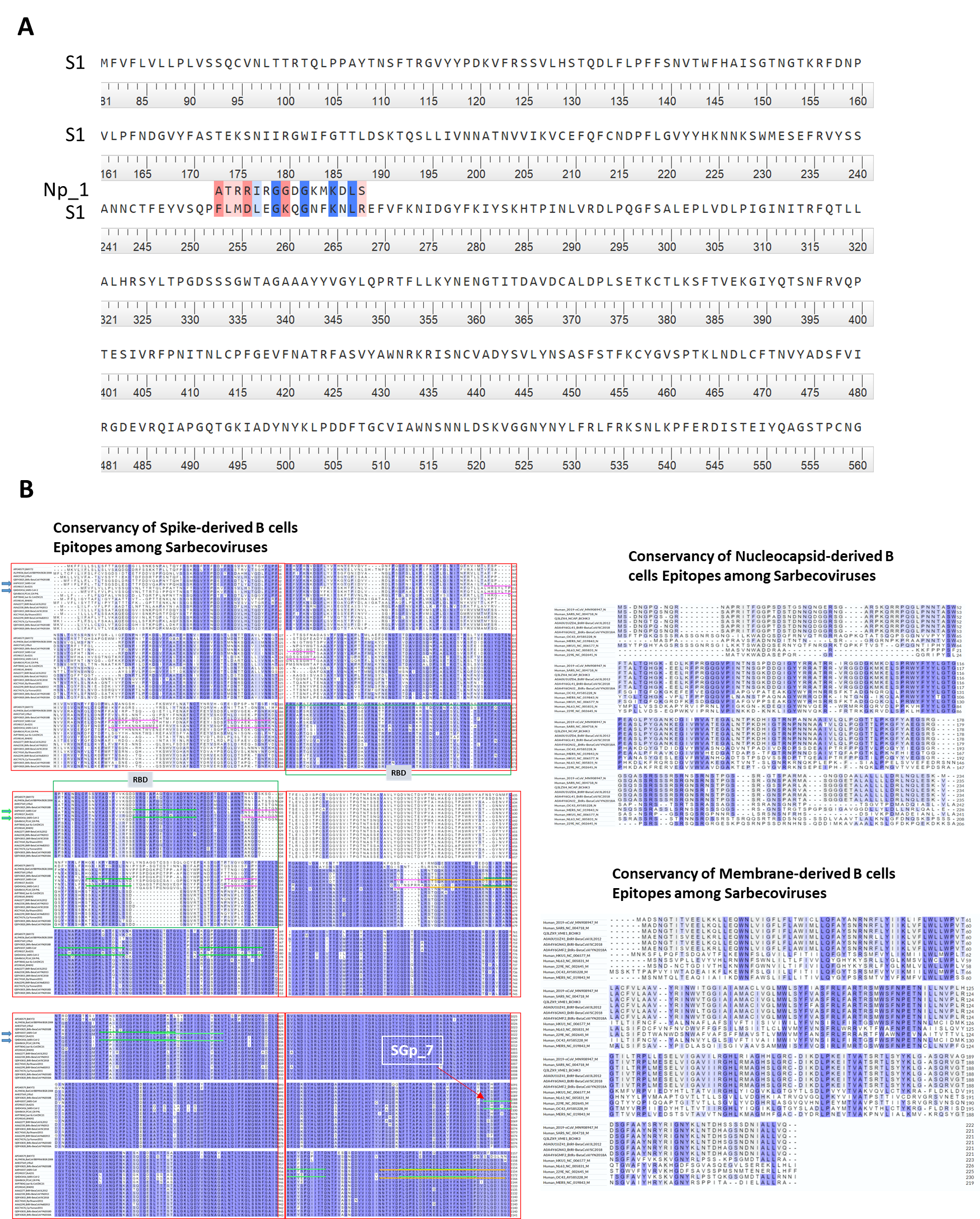


**Figure S4. The mouse sera from each three peptides (SGp_7, SGp_10, CSNP4) inhibit all SARS-CoV-6 variants of concerns (VOCs) pseudovirus infection in hACE2-293T cells.** (A, B, C) Alpha, Beta, Gamma, Wu01, Delta, and Omicron Spike pseudovirus-infected hACE2-293T cells were treated with three antibodies from mouse sera immunized with the three peptides at varying dilutions (1:200, 1:100, and 1:50), and their luciferase reporter activities were measured. Data are shown as mean ± STD (compared with control vs *p < 0.05; **p < 0.01; ***p < 0.001; Student’s t test).


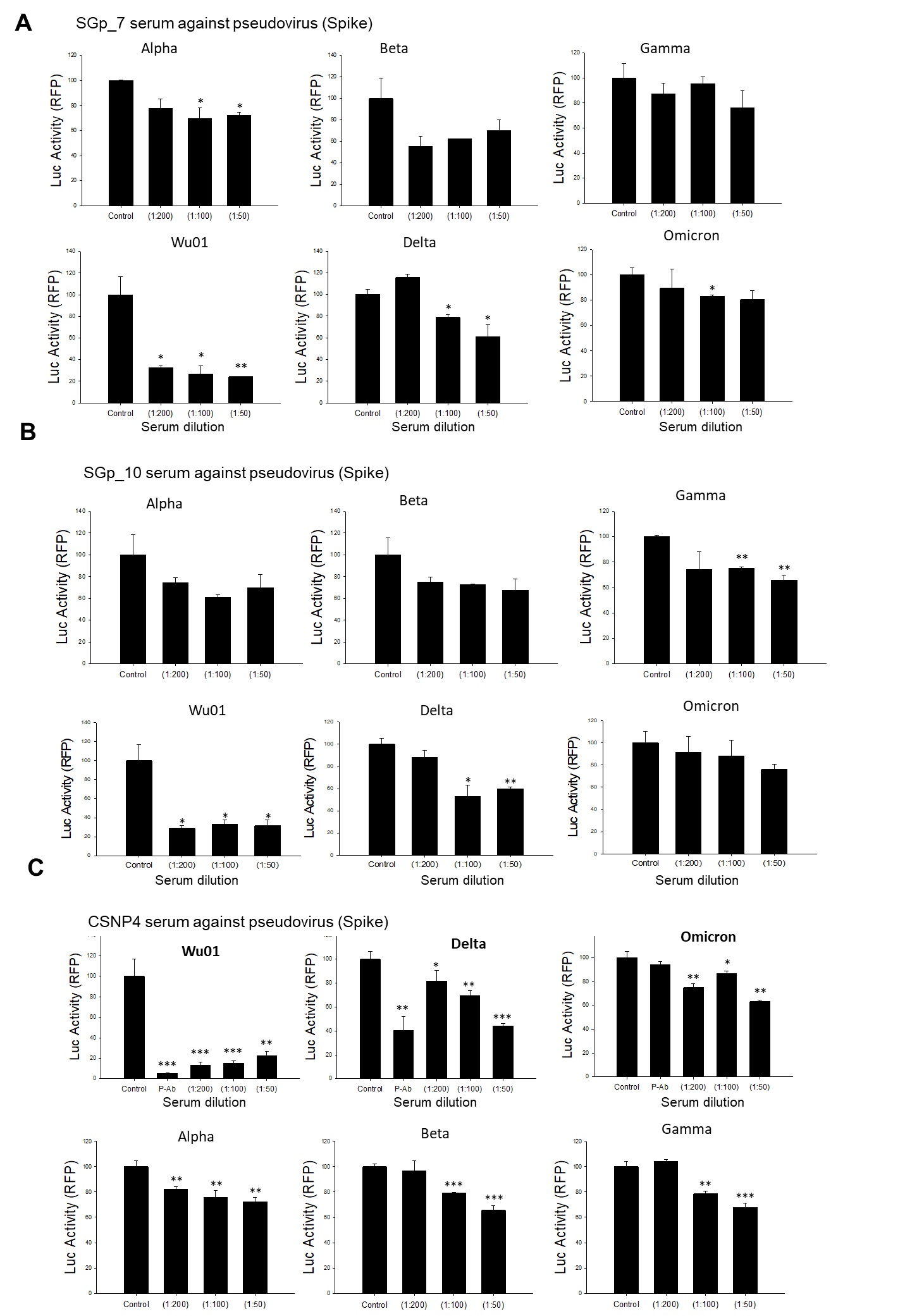


**Figure S5. Structural and Binding Analysis of HLA-DRB1 Variants and CII Peptides**. A) Multiple sequence alignment of the beta chains of the HLA-DRB1 (top panel) and theirs superimposed structure showing the variant residues in Box1 and Box2. B) Among CII-1, CII-2, and CII-4 peptides, CII-1 and CII-2 favorably bind QA1*05:01/QB1*03:01, QA1*01:02/QB1*05:01, respectively. C) CII-2: AGNGGDAALALLLLDRLNQL, binds DRB1*15:01, DRB1*09:01, and DQA1*01:02/DQB1*05:01 by anchoring Leucine into their P1, P4, P6 and P9 pockets.


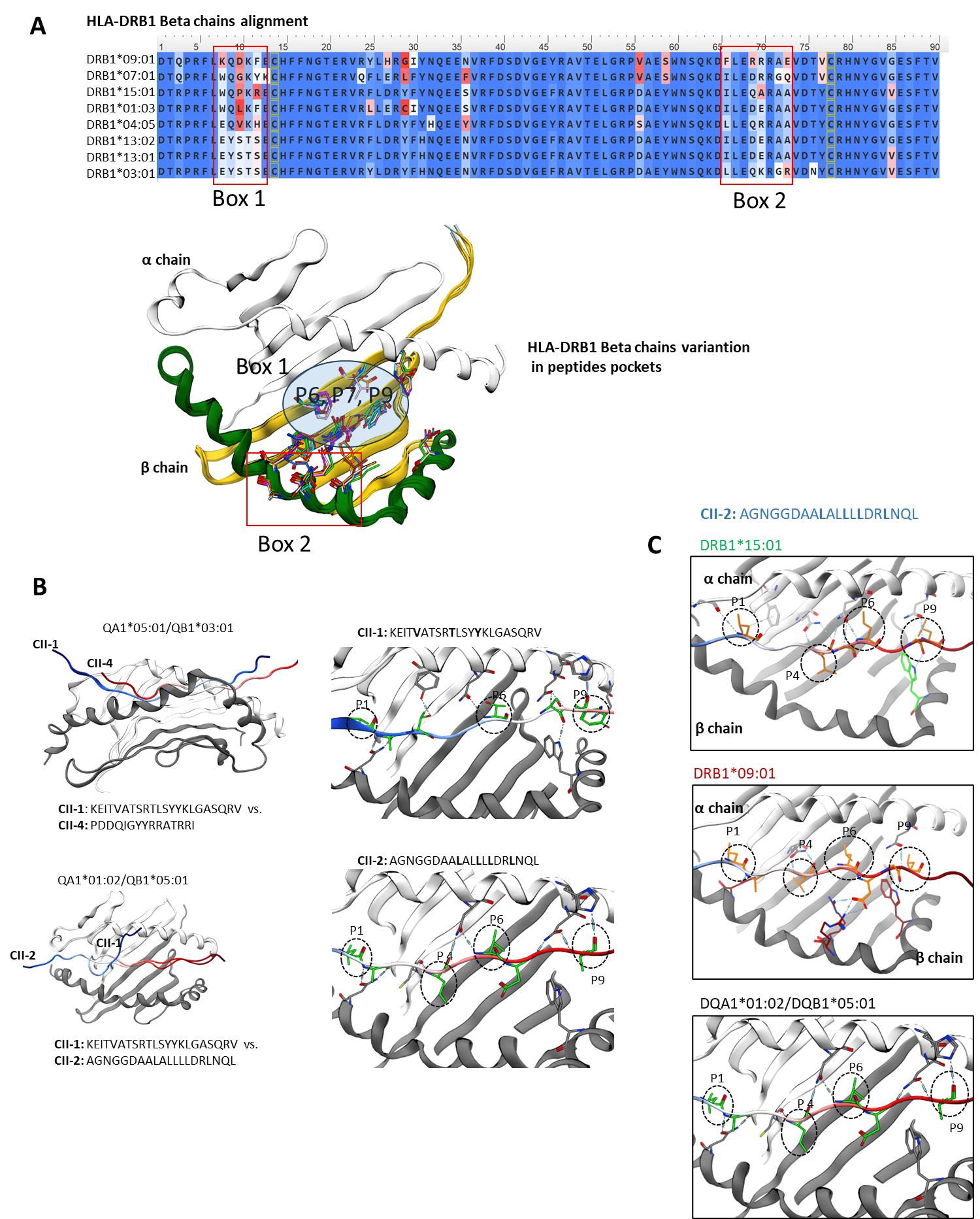


**Figure S6. Evaluation of IFN-γ Response in PBMCs and Splenocytes Stimulated with SARS-CoV-2 S1 Protein and Peptide Candidates.** A) PBMCs were isolated from blood of 3 vaccinated healthy donors and expanded with CD3 and CD28 for 9-14 days. B) S1 protein and peptide candidates, 9 for HLA class I & 5 for HLA class II were treated with expanded PBMC for 48 hrs. IFN-γ was measured to confirm the T-cell response. *P < 0.05, Kruskal-Wallis multiple comparisons test was used to assess the positive value. C) Lymphocytes were isolated from the spleens of non-immunized mice (n = 2 mice), seeded, and then incubated for 48 hours by stimulating with peptides or S1 proteins. The IFN-γ levels were detected using an IFN-γ ELISA detection kit. Data are presented as mean ± standard deviation (compared with NC vs. *p < 0.05; **p < 0.01; ***p < 0.001; Student’s t-test).


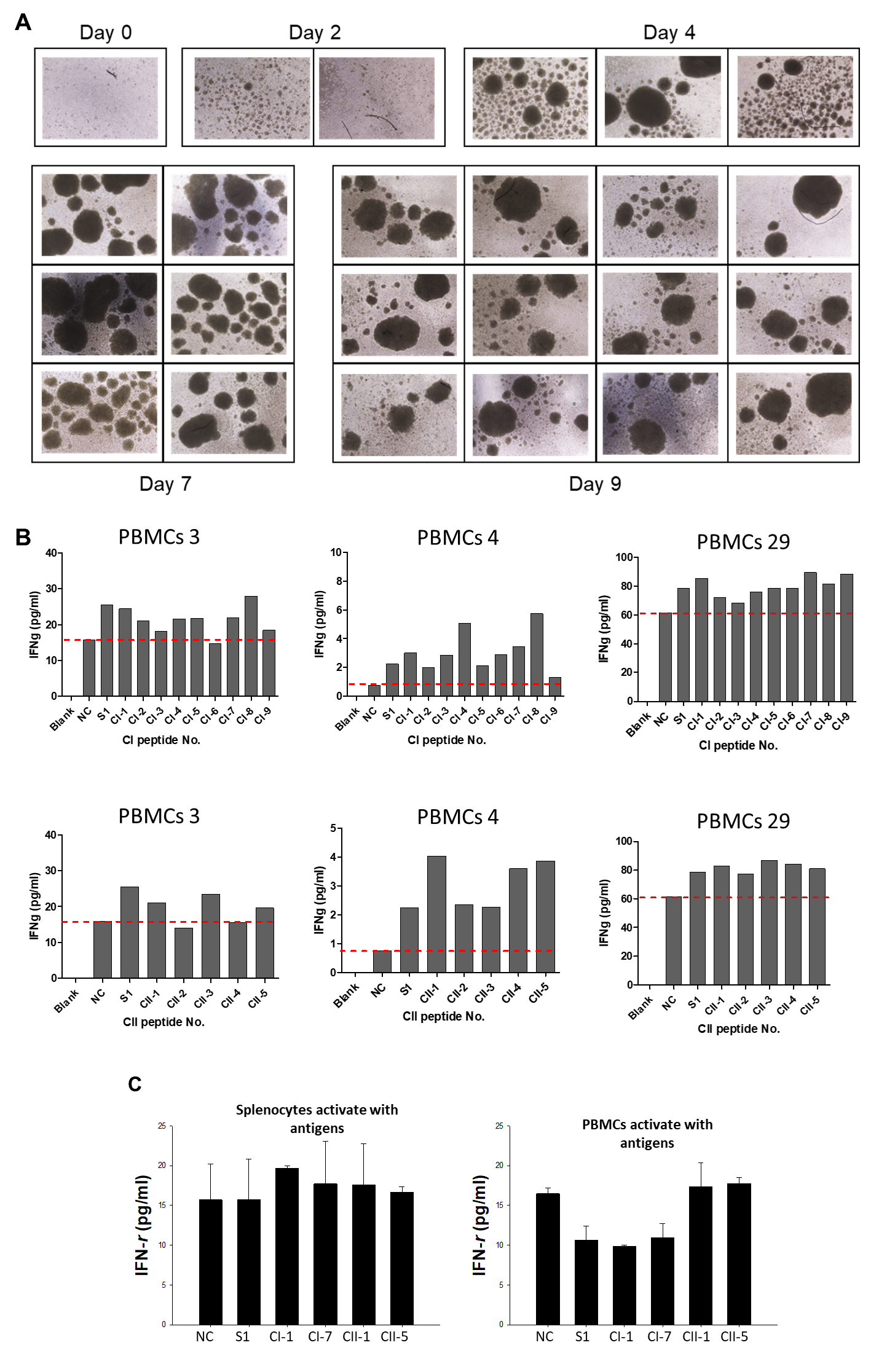


**Figure S7. Immunocytochemical Analysis of CD4, CD8, CD25, and CD44 Marker Expression in Splenocytes Stimulated with T-cell Peptides.** Splenocytes isolated from T-cells peptides immunized mice were stimulated (Stimul) with T-cell peptides and analyzed for CD4, and CD8 markers (A) and CD25 and CD44 markers (B) expression using immunocytochemistry. This data corresponds to that shown in figure 6 F and G.


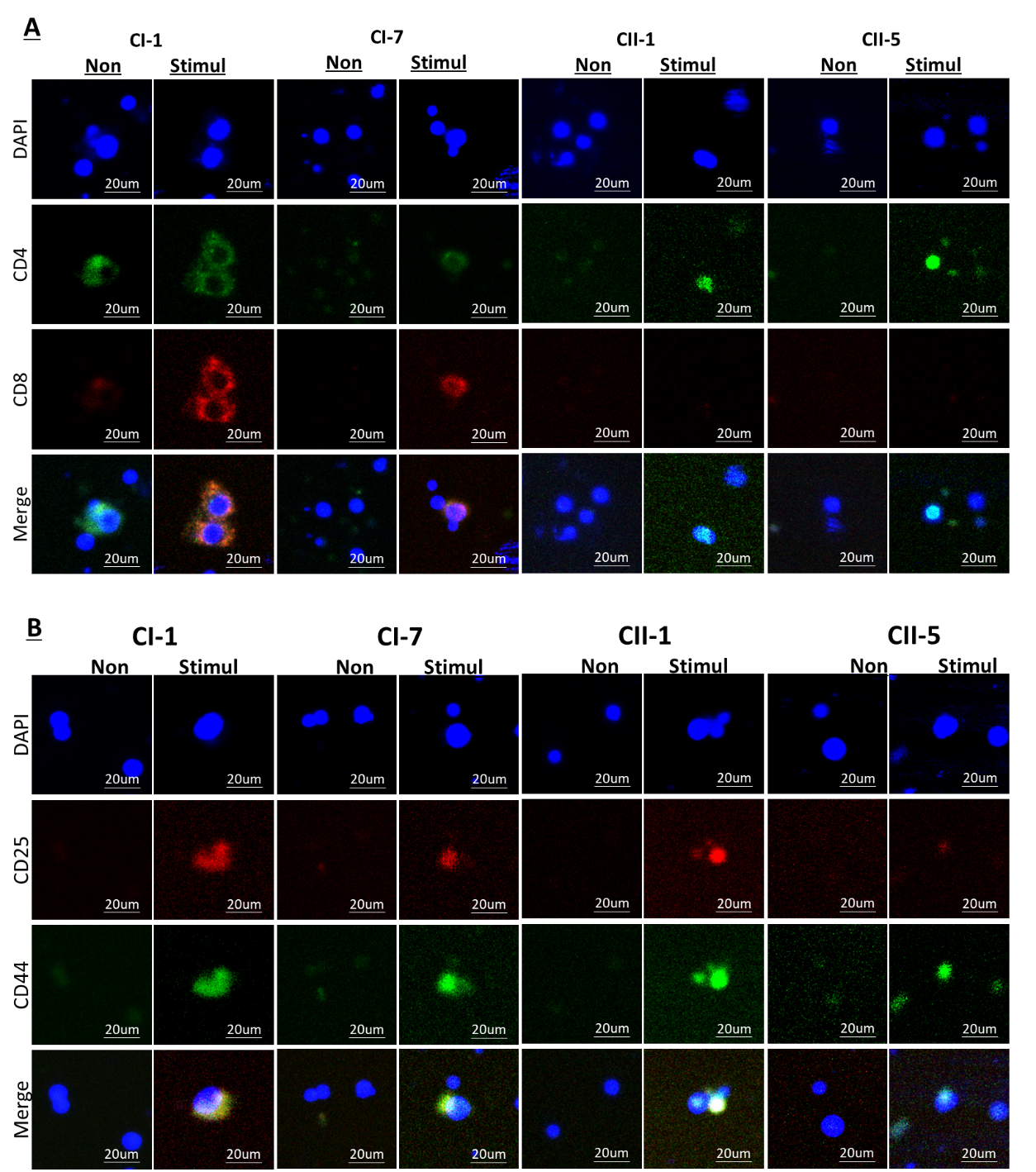


**Supplementary Tables**

**Table S1. List of the B cell peptides and their AlpaFold 2 predicted models.**


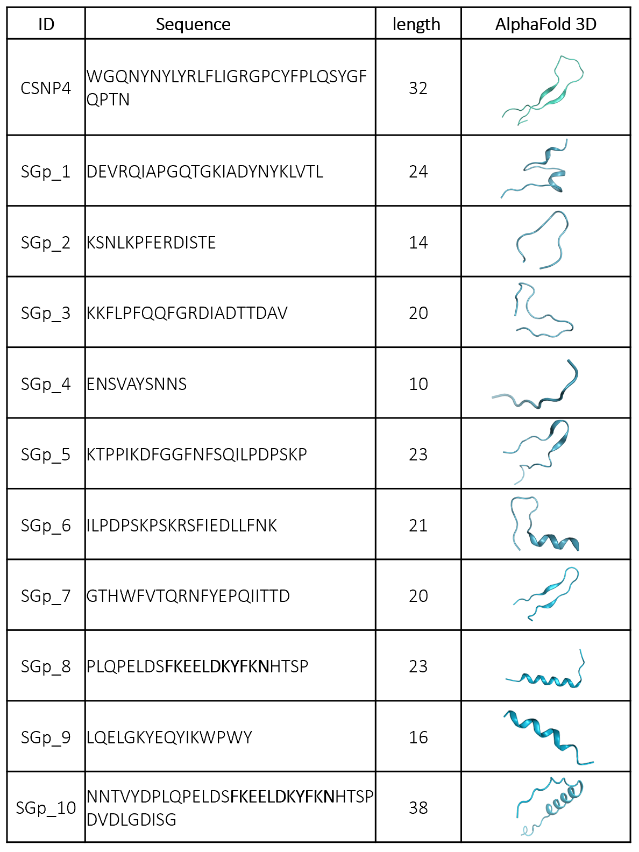


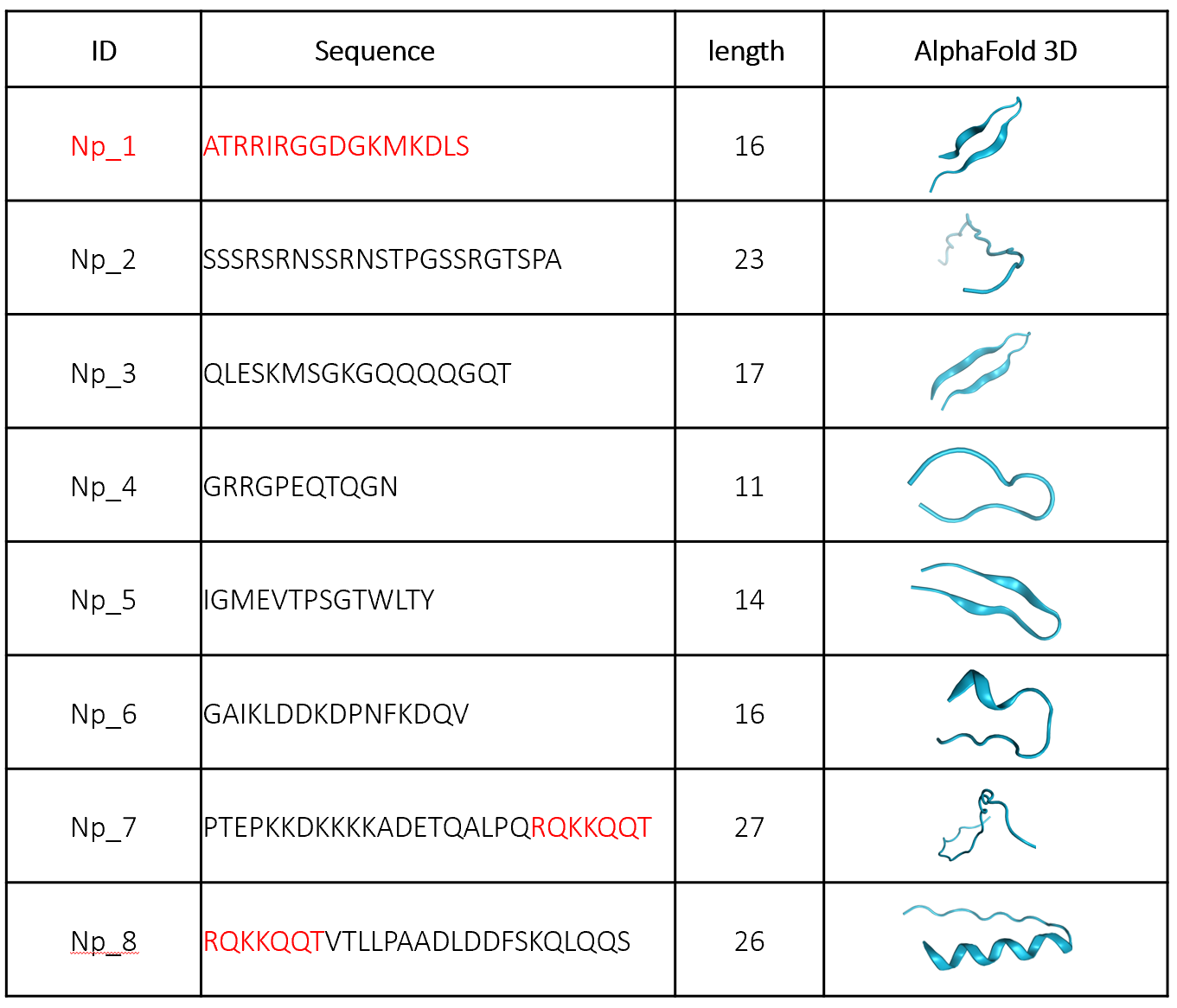


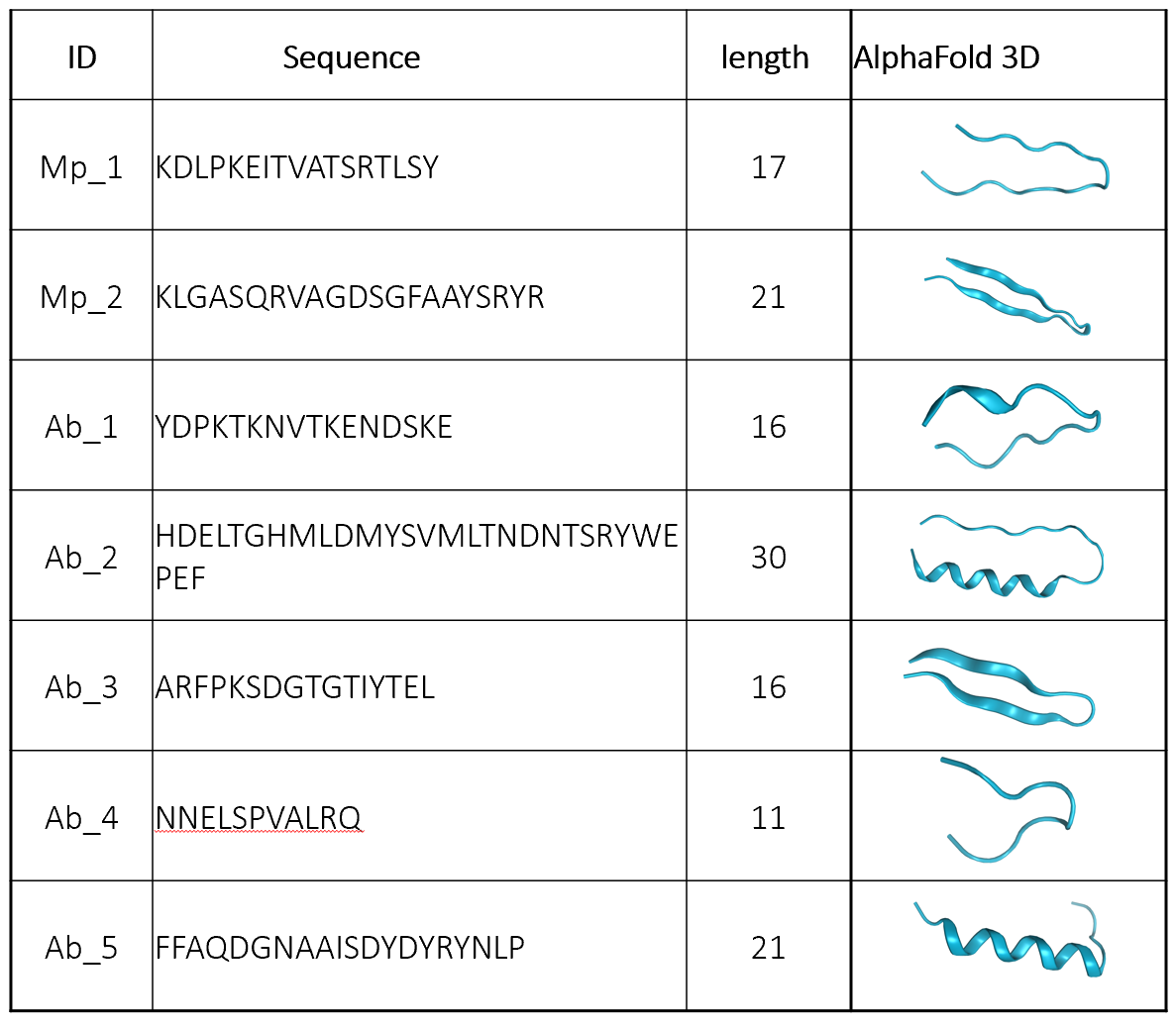


**Table S2: List of the MHC class I peptides. Peptide with Exp_ID were evaluated for IFN-γ assay using human PBMCs.**

|  | **ID** | **Exp_ID** | **Sequence** | **Source Prot** |
| --- | --- | --- | --- | --- |
|  | Control |  | LLLDRLNQL | Nucleocapsid |
| 1 | 0094-Ajou2-KY | CI-9 | KRVDWTIEY | 1ab |
| 2 | 0101-Ajou2-AL |  | AEIVDTVSAL | 1ab |
| 3 | 0103-Ajou2-KW | CI-8 | KSAGFPFNKW | 1ab |
| 4 | 0080-Ajou2-KV |  | KLLEQWNLV | Membrane |
| 5 | 0084-Ajou2-FI |  | FVLAAVYRI | Membrane |
| 6 | 0093-Ajou2-VY |  | VATSRTLSY | Membrane |
| 7 | 0105-Ajou2-LA |  | LSYYKLGASQRVA | Membrane |
| 8 | 0085-Ajou2-LL | CI-2 | LQLPQGTTL | Nucleocapsid |
| 9 | 0092-Ajou2-KF | CI-7 | KAYNVTQAF | Nucleocapsid |
| 10 | 0096-Ajou2-GW |  | GANKDGIIW | Nucleocapsid |
| 11 | 0098-Ajou2-KK |  | KTFPPTEPKK | Nucleocapsid |
| 12 | 0099-Ajou2-QF |  | QFAPSASAF | Nucleocapsid |
| 13 | 0100-Ajou2-ML |  | MEVTPSGTWL | Nucleocapsid |
| 14 | 0081-Ajou2-LL |  | LLYDANYFL | ORF3a |
| 15 | 0089-Ajou2-FK |  | FTIGTVTLK | ORF3a |
| 16 | 0102-Ajou2-II |  | IPYNSVTSSI | ORF3a |
| 17 | 0104-Ajou2-LY | CI-5 | LLKEPCSSGTY | ORF7a |
| 18 | 0079-Ajou2-DV |  | DEFVVVTV | ORF9b |
| 19 | 0095-Ajou2-AL | CI-6 | AEIRASANL | Spike |
| 20 | 0082-Ajou2-NV | CI-1 | NATNVVIKV | Spike |
| 21 | 0083-Ajou2-HW |  | HADQLTPTW | Spike |
| 22 | 0086-Ajou2-RK |  | RLFRKSNLK | Spike |
| 23 | 0087-Ajou2-TK |  | TLKSFTVEK | Spike |
| 24 | 0088-Ajou2-VK | CI-3 | VTYVPAQEK | Spike |
| 25 | 0090-Ajou2-QI | CI-4 | QYIKWPWYI | Spike |
| 26 | 0091-Ajou2-VF |  | VYSTGSNVF | Spike |
| 27 | 0097-Ajou2-ML |  | MIAQYTSAL | Spike |

**Table S3: List of the MHC class II peptides. Bold were evaluated for IFN-G assay using human PBMCs**

| **ID** | **Exp_ID** | **Sequence** | **Source Prot** | **HLA-DRB1** | **HLA-DQ** | **HLA-DP** |
| --- | --- | --- | --- | --- | --- | --- |
| **ClassII-2** | **CII-1** | **KEITVATSRTLSYYKLGASQRV** | Membrane | *01:03; *09:01, *15:01, *13:02 | QA1*01:02/B1*05:01  QA1*05:01/B1*03:01 | A1*01:03/B1*04:01  A1*01:03/B1*02:01 |
| **ClassII-3** | **CII-2** | **AGNGGDAALALLLLDRLNQL** | 1ab | *03:01; *09:01; *15:01; *13:02 | QA1*01:02/B1*05:01 | A1*01:03/B1*04:01  A1*01:03/B1*02:01 |
| **ClassII-4** | **CII-3** | **LMIERFVSLAIDAYP** | 1ab | *04:05; *09:01; *15:01 |  |  |
| **ClassII-9** | **CII-5** | **QFAYANRNRFLYIIK** | Membrane | *09:01; *15:01; *13:01; *13:02 |  |  |
| **ClassII-8** | **CII-4** | **PDDQIGYYRRATRRI** | Nucleocapsid | *09:01; *15:01 | QA1*05:01/B1*03:01 |  |
|  |  |  |  |  |  |  |

Table S4: Murine MHC-binding prediction of the CII-1 and CII-5 peptides.


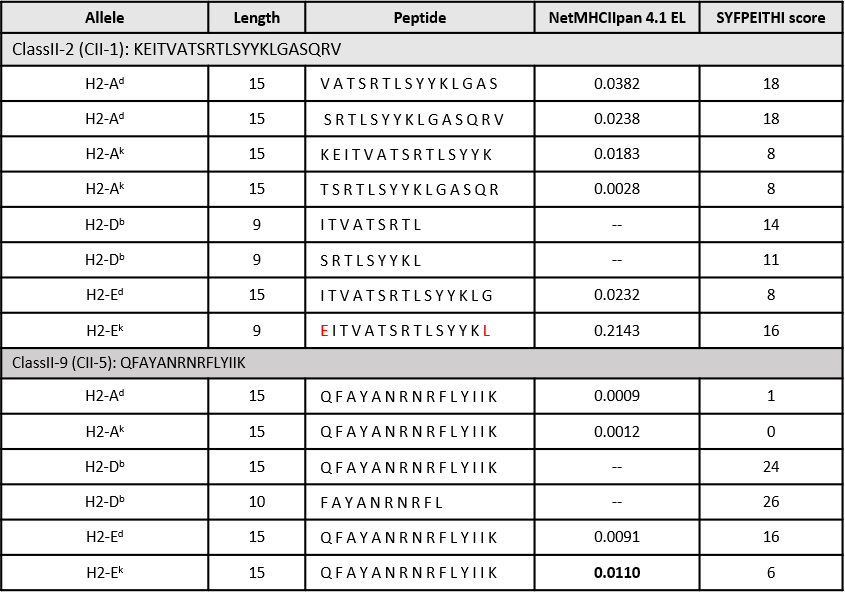

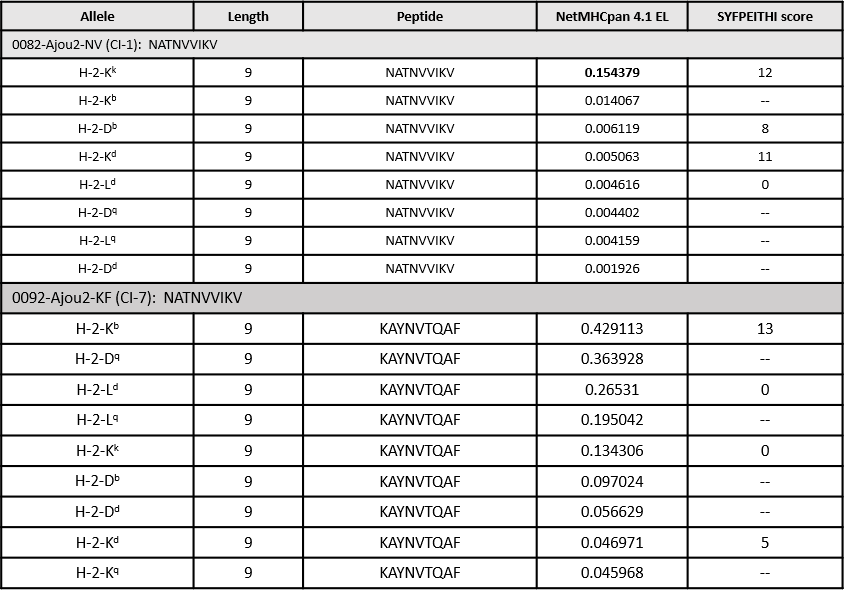

Supplement: Supplementary file 1 — Supporting Information [file ADVS-12-2409919-s001.docx]
